# *In vivo* cellular-resolution imaging of retina: modality, cells, and clinical implications

**DOI:** 10.3389/fopht.2025.1682303

**Published:** 2026-01-13

**Authors:** Shaohua Pi, Richard Brown, Samuel Yun, Lingyun Wang

**Affiliations:** 1 Department of Ophthalmology, University of Pittsburgh, Pittsburgh, PA, United States; 2 University of Pittsburgh Medical Center (UPMC) Vision Institute, University of Pittsburgh, Pittsburgh, PA, United States; 3 Department of Bioengineering, University of Pittsburgh, Pittsburgh, PA, United States

**Keywords:** adaptive optics, cellular imaging, *in vivo* imaging, microglia, ophthalmoscopy, optical coherence tomography, photoreceptors, retina

## Abstract

The retina, a crucial component of the human eye for vision, is responsible for converting light signals into neural signals that the brain can interpret. It’s a complex tissue, rich in photoreceptors, and supported by various other cell types, including inner nuclear layer cells, ganglion cells, pigmented epithelial cells, immune cells, and vascular cells. Each of these cells plays a vital role in visual processing and understanding of their function and interactions are essential for assessing vision health and diagnosing diseases. Traditionally, studying the retinal cells has relied heavily on histological techniques, which, despite their utility, offer only static images and require invasive procedures that preclude the observation of dynamic biological processes. In this context, recent advancements of *in vivo* imaging technologies have marked a significant leap forward. Techniques such as ophthalmoscopy, optical coherence tomography (OCT), adaptive optics (AO), two-photon excitation microscopy (TPM), and light-sheet fluorescence microscopy (LSFM) now enable the direct observation of retinal cells in living organisms. This shift from invasive, static methods to dynamic, non-destructive imaging allows for a more nuanced understanding of retinal cell behavior under physiological conditions. It opens up new avenues for the study of the retina’s complex ecosystem in both health and disease, facilitating early diagnosis of retinal conditions and offering new strategies for treatment. By offering a window into the live retina, *in vivo* imaging stands as a cornerstone of contemporary ophthalmology, promising to enhance our understanding of eye health and to spur innovations in the diagnosis and treatment of ocular diseases.

## 1 Introduction

Visual processing begins with light passing through the cornea and the lens, projecting onto the retina; transporting neural transduction of these light signals via the optic nerve to the cerebral cortex (1–4). Among them, the retina is a thin layer of tissue and shares an embryonic origin with the central nervous system (1). In the retina, the visual circuit involves light sensing by photoreceptors (3, 5–8), initial processing by cells in inner nuclear layer (INL) such as bipolar cells (4, 9) and amacrine cells (10), and feature extraction by retinal ganglion cells (RGCs) (11). Additionally, these processes are supported by cells in retinal pigment epithelium (RPE) (12–14), immune system (15–18), and vasculature (19, 20). In recent years, there has been growing recognition that many retinal diseases begin with dysfunction or loss at the cellular level long before traditional clinical imaging can detect structural abnormalities. Photoreceptor stress (21), RPE metabolic changes (22, 23), early microvascular instability (24), and activation of microglia (25) and Müller glia (26, 27) often precede measurable retinal thinning or visual decline.

With micron- or submicron- resolution provided by confocal microscopy and super-resolution microscopy in histological tissue examinations from animal models or humans, retinal cells and their ultra-structures can be revealed to gain knowledge of cellular function (28, 29). Immunostaining techniques can highlight specific targets to further improve visualization (30) and delineate between the cells and organelles (31–33). Furthermore, electron microscopy (EM), along with its various iterations, can visualize retinal structures at the nanometer level (29, 34). However, these imaging modalities are subjected to drawbacks including static snapshot, destructive process, time-consuming sample preparation, artifacts due to processing, and ethical and practical constraints.

*In vivo* imaging techniques are superior by overcoming these challenges, allowing longitudinal monitoring of ocular disease states across weeks and months, providing information about natural disease course, aging, and treatment efficacy both in research and clinics (9, 19, 35, 36). The history of *in vivo* ophthalmic imaging dates to the invention of the first ophthalmoscopy by Hermann Von Helmholtz (37, 38). Since that time, generations of physicians and engineers have improved upon his design to advance in this field. Digital fundus photography captures the true color image of the retina for quantitative analysis (39–41). Later, scanning laser ophthalmoscopy (SLO) offers higher contrast than fundus photography due to its capability to reduce scattering effects and allows for evaluation of fundus autofluorescence (42). Additionally, indocyanine green angiography (ICGA) and fluorescein angiography (FA) reveals the retinal and choroidal vasculature, both of which have advanced ophthalmologists’ understanding of retinal diseases (43–48). Further, the invention of optical coherence tomography (OCT) in the 1990s revolutionized ophthalmic imaging and quickly became an indispensable tool for all retinal clinicians and researchers (49). Retinal laminar tissue, blood vessels, and various lesions such as thickness thinning, edema, leakage, and neovascularization can now be readily examined in patients to assist in the diagnosis and management of ocular diseases (50–54).

Conventional modalities provide invaluable macroscopic views but remain limited in their ability to resolve individual cells or to monitor subtle cellular events that drive disease progression. The challenges preventing the visualization of retinal cells *in vivo* include limited aperture and resolution, motion due to breath and heartbeat, light safety concerns, and aberrations generated by the eye and the optical system (55–57). Recent technological advances in hardware and post-processing offer advantages in overcoming these difficulties towards cellular resolution retinal imaging (36, 58–63). Emerging technologies now enable *in vivo* visualization of individual photoreceptors (59, 64), ganglion cells (65, 66), RPE cells (64, 67), immune cells (68, 69), and microvascular elements (70, 71), offering unprecedented insight into disease onset and dynamics. Cellular-resolution imaging has already begun to show clinical relevance: adaptive optics (AO) can quantify photoreceptor integrity in inherited retinal diseases (72); visible light OCT (vis-OCT) provides enhanced layer contrast and enables retinal oximetry (73); full field OCT (FF-OCT) (74) can detect early microstructural abnormalities; and dynamic contrast OCT (DyC-OCT) (75, 76) and two-photon excitation microscopy (TPM) (77) have revealed functional cellular responses previously accessible only through histology or animal models. These capabilities open new pathways for early diagnosis, monitoring treatment response, evaluating neuroprotective therapies, and understanding the mechanisms underlying disorders such as age-related macular degeneration (AMD), diabetic retinopathy, glaucoma, and optic neuropathies. Thus, the transition from tissue-level to cellular-level retinal imaging represents a crucial evolution in both research and clinical care, providing the opportunity to detect disease earlier, stratify risk more precisely, and evaluate therapeutic efficacy at the level where pathology originates. In this review, we will highlight recent developments in *in vivo* retinal imaging techniques. A general overview of emerging imaging modalities for *in vivo* cellular imaging of retina are summarized in Section 2. Following that, we introduce studies on individual retinal cell types in Section 3, including the photoreceptors, INL cell bodies, RGCs, as well as supporting cells such as RPE cells, immune cells, blood cells. Finally, we discuss the potential clinical impact of these emerging technologies for the early diagnosis of retinal diseases in Section 4. We hope this review will provide perspectives for this exciting field and facilitate advancements in widespread clinical adoption.

## 2 General overview of imaging modalities

### 2.1 Scanning laser ophthalmoscopy

Ophthalmoscopy is crucial in examining the retina and optic disc; it has undergone continuous advancement since its inception and is readily utilized in ophthalmology clinics. Fundus photography was first commercially produced by Carl Zeiss in 1926 (78). Hansell and Beeson first proposed a new compact xenon arc lamp (FA5) in the Zeiss-Nordenson retinal camera to provide the existing system with an improved light source (79). Nevertheless, the optical and mechanical complexity of early devices limited their clinical utilization. With the development of the hand-held and digital fundus camera, contemporary ophthalmologists can easily capture retinal images with high resolution to assist the diagnosis of eye diseases (80–83).

**Figure 1:**
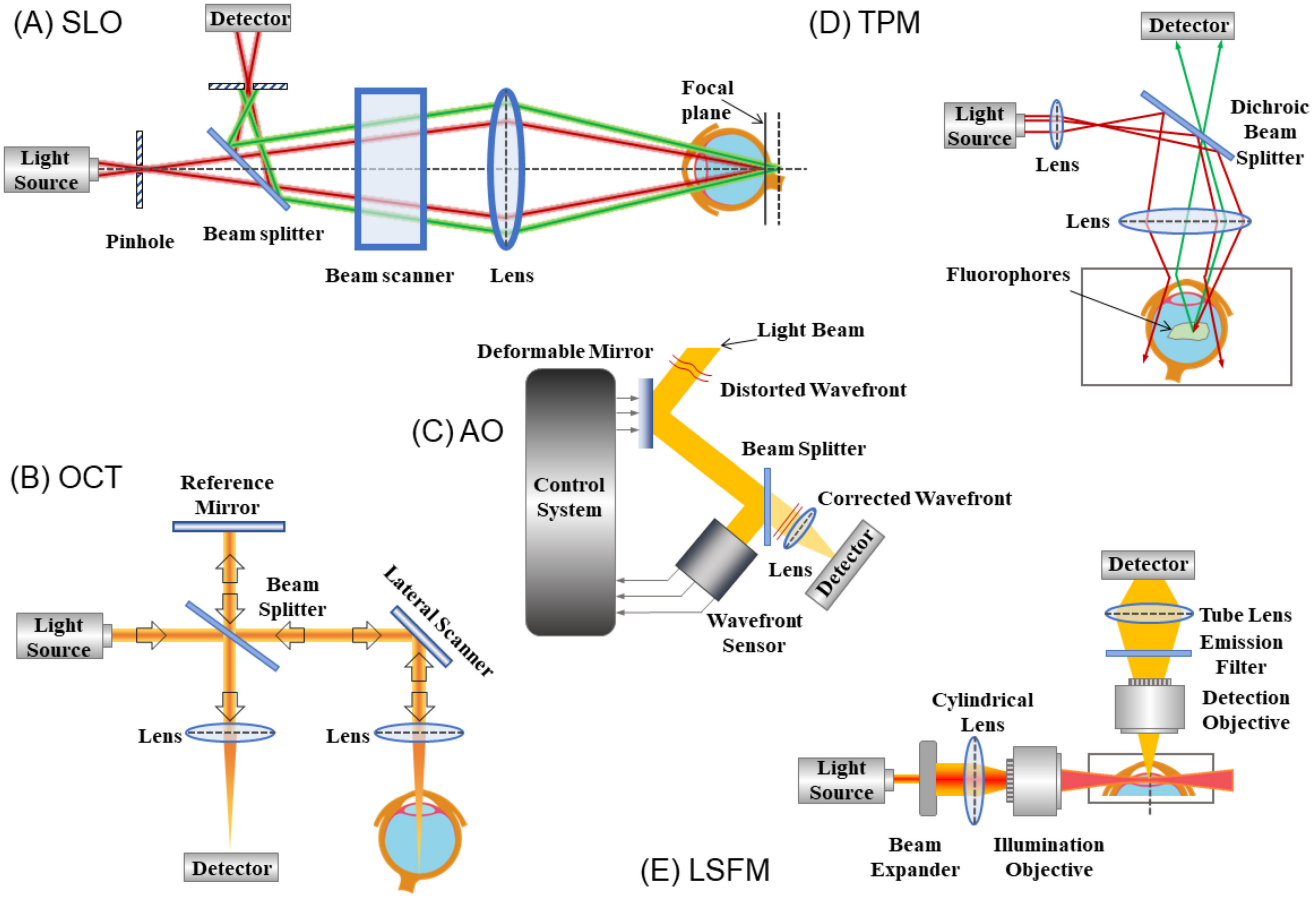
Schematic of the principles of five imaging modalities: **(A)** Scanning Laser Ophthalmoscopy, SLO; **(B)** Optical Coherence Tomography, OCT; **(C)** Adaptive Optics, AO; **(D)** Two-photon Excitation Microscopy, TPM; **(E)** Light-sheet Fluorescence Microscopy, LSFM.

SLO was first demonstrated by Webb et al. in 1981 (**Figure 1A**) (84); however, its non-confocal design suffered from loss of contrast because it accepted all reflected light (85). In 1997, Webb et al. presented the principles of confocal SLO (cSLO) that provided a higher contrast view of the fundus (86). Confocal SLO uses a highly collimated narrow beam of light in a small region to sweep across the retina. The confocal aperture (pinhole) minimizes scattered reflected light by allowing only focused light to reach the photodetector. The applications of cSLO with its high-resolution include identifying early glaucoma cases (87), examining choroidal circulation (88), and detecting retinal ganglion cell damage (89).

Although SLO enjoys great popularity in ophthalmology, it also faces challenges for *in vivo* imaging. Eye motion poses a significant problem, making correction of motion distortions necessary (90). To date, many eye tracking techniques have been reported (91–93). Compared with the fundus camera, another limitation of SLO is its monochrome images. Although methodologies combining three wavelengths (red, green and blue) have been used to generate color SLO images (94), image quality is reduced due to significant loss of light.

### 2.2 Optical coherence tomography

OCT is an efficient and non-invasive imaging technique which can provide high-resolution cross-sectional images of biological tissues (49). It is based on the principle of Michelson interferometry, utilizing interference between the light beam passing through the sample (in this case the retina) and a reference beam to generate images (**Figure 1B**). The advantages of OCT, namely its high-resolution sectioning and high contrast imaging abilities, enable its application in many clinical fields such as cardiology, dermatology, gastroenterology, and ophthalmology (95–100).

OCT methods can be classified into two types, time domain (TD)-OCT and Fourier domain (FD)-OCT (101). When OCT was first reported by Huang et al. in 1991, time-domain detection was used to image *ex vivo* retinas and coronary arteries (49). FD-OCT, including spectral domain (SD)-OCT and swept-source (SS)-OCT (102, 103), features a relatively higher scanning speed and eliminates the need for depth scanning (104). SD-OCT measures all wavelengths of light simultaneously using a spectrometer, allowing for a higher sensitivity than TD-OCT and faster scanning speeds (29,000 to 80,000 A-scans per second) (105). Compared to SD-OCT, SS-OCT features a source modulation scheme to achieve the wideband light detection with narrow-linewidth, frequency-swept laser (106). The Fourier domain mode locking (FDML) laser enables SS-OCT to achieve an incredible A-scan rate of up to 3.35 MHz at 1060nm, thus enabling the capture of transient activities at high volume rate (107).

To improve sensitivity and specificity of diagnosis, numerous endeavors have been made by researchers to achieve higher resolution in OCT. The broadband light source, like multiplexed super luminescent diodes (SLD), improves the axial resolution of OCT, according to the principle of OCT imaging (108–110). For instance, Drexler et al. presented ophthalmic OCT systems that could image retinal and corneal morphology with an axial resolution of 2-3 µm based on broadband Ti: Al_2_O_3_ laser (111) and achieved the theoretical axial resolution of 1 µm in biological tissue by using a Kerr-lens mode-locked Ti: sapphire laser in TD-OCT (112). Later, researchers developed high-speed ultrahigh-resolution OCT (UHR-OCT) systems by combining FD-OCT techniques and broadband lasers as FD-OCT significantly improves the sensitivity of OCT and imaging speed (105, 113). For example, Wojtkowski et al. demonstrated an UHR-FD-OCT with an axial resolution of 2.1 µm in tissue and 16,000 axial scans per second (105). In 2017, Werkmeister et al. developed an UHR-OCT system with the 1.2 µm × 20 µm (axial × transverse) resolution for human corneal imaging (114).

Different from axial resolution, it is difficult to increase the transverse resolution of OCT because there exist trade-offs between the transverse spot size and axial focal range, aberrations, and ranging depth (115). Hence, more work related to focus extension (116) and aberration correction (117) needs to be conducted to improve the imaging performance of OCT. Besides the AO to correct aberration, which will be introduced in next section, full-field OCT (FF-OCT) demonstrates an important solution in achieving high transverse resolution (118, 119). previously, it was primarily limited to *ex vivo* samples (120–122). Recently, with the improvement of cameras and other advancements, researchers were able to acquire the cellular retinal imaging *in vivo* in humans with the FF-OCT (123). Efforts are continuously made to push FF-OCT imaging reliable to be more suitable for clinical studies in patients in terms of real-time, high-sensitivity, and large field of view (74, 124–126).

Most OCT devices use near-infrared (NIR) light. However, OCT using visible light (vis-OCT) may offer extra benefits. Vis-OCT was first reported in 2002 (127). Compared with NIR-OCT, it has better axial resolution because the axial resolution has quadratic dependence on the center wavelength of the light source. For instance, Lichtenegger et al. achieved an axial resolution of 0.88 μm in brain tissue using a broad visible light spectrum (425–685 nm) (128). On top of that, the visible spectral range of vis-OCT makes it suitable for detecting biological tissues at shorter wavelengths. Nowadays, vis-OCT has been applied to image human eye. Yi et al. demonstrated the first human retinal imaging using vis-OCT, and the results show that vis-OCT has higher contrast for the photoreceptor inner and outer segment (IS/OS), the outer segment of photoreceptors (OS), and the retinal pigmented epithelium (RPE) imaging than NIR-OCT (129). Chen et al. proved the feasibility of vis-OCT oximetry in humans and increased its accuracy by using the statistical-fitting approach (130). Recently, Yi’s team achieved the first vis-OCT angiography for human retinal imaging, and they were capable of measuring sO_2_ in vessels with diameter smaller than 100 µm (131).

Another new development capable of providing high-contrast imaging between cellular structures is dynamic contrast OCT (DyC-OCT). DyC-OCT is a label-free method that detects temporal variations in light signal intensity to illustrate changes in cellular activity and motion, producing functional maps of live cells and tissues across a wide range of timescales (132, 133). Uses of DyC-OCT include visualizing cell and tissue morphology by highlighting regions of high temporal signal variation (134), and assessment of cell viability by monitoring cellular responses to physical and chemical stimuli (135). However, most applications of DyC-OCT currently utilize ex vivo living tissues as the repetitive scanning needed for 3D DyC-OCT limits *in vivo* usage due to the presence of motion artifacts (133). The use of parallel OCT methods or machine-learning algorithms can be used to significantly reduce imaging time (133).

### 2.3 Adaptive optics

AO is a technology that was initially developed in astronomy to compensate for the blur-inducing aberrations caused by atmospheric turbulence (136). Since the first AO system was used in retinal imaging (137), AO has gained popularity in ophthalmology due to its ability to sharpen retinal images previously blurred by ocular aberrations (138). A conventional AO system for retinal imaging consists of three essential components: a wavefront sensor, a corrective element, and a control system (**Figure 1C**) (59). The wavefront sensor measures the ocular aberrations, which signals the AO control system to modify the corrective element to cancel out aberrations.

AO has been successfully combined with fundus cameras, scanning laser ophthalmoscopy, two-photon excitation microscopy, and optical coherence tomography (137, 139–141). The invention of Hartmann-Shack wavefront sensor and the deformable mirror (DM) made it possible for AO flood-illumination ophthalmology (AO-FIO) to perform single-cellular imaging *in vivo* of cone cells (137, 142). In 1997, Liang et al. reported the first AO fundus camera, which had the capability of imaging microscopic structures the size of single cells in the retina (137). Since that time, AO boost modality has been used to observe the microcystic changes in the inner retina of patients (143), visualize the vasculature in living human retina (144), and image foveal cones and rods (145). With the help of the wavefront sensor and the DM, Roorda et al. invented the first AO-SLO in 2002, which had a higher imaging quality than AO-FIO (140). AO-SLO has been widely used in the diagnosis of patients with eye diseases (146). Compared to OCT, the axial resolution of FIAO and AO-SLO is relatively low. Therefore, the combination of AO technology and OCT is essential, because it can achieve a higher axial resolution (below 3 µm) (139). In 2004, Hermann et al. combined AO and TD-OCT to improve the signal-to-noise (SNR) of the system up to 9 dB (147). Due to the high axial resolution of AO-OCT, it can provide detailed images of inner retinal layers (139, 147). Recently, AO has been combined with OCTA, generating significantly reduced shadowing artifacts of the inner retinal vasculature (148). Additionally, Zhang et al. have integrated OCT into an existing AO-SLO system for *in vivo* imaging of mice retina capable of providing a ~6 µm axial resolution for AO-OCT and ~1 µm lateral resolution for AO-SLO-OCT (149).

Recent developments in AO utilize machine learning to further clean up image. Zhou et al. created two semi-supervised models, RGC-CCT and RGC-CPS, capable of identifying ganglion cells from AO-OCT volumes with minimal manual annotation (150). Additionally, P-GAN, a separate model developed by Das et al., is capable of extracting RPE cell features muddied by speckle noise from a single AO-OCT scan, significantly reducing imaging time while preserving cellular imaging accuracy (151). Integrating AI in the post-processing pipeline provides promise for expanding AO imaging into clinical use by reducing scan acquisition time and mitigating operator dependency.

AO-based retinal imaging technology shows great potential in clinical utility (152–155). However, the combination of AO and ophthalmic modalities significantly increases cost and system complexity, which presently limits the commercialization of these systems.

### 2.4 Two-photon microscopy

Two-photon microscopy (TPM) provides large depth penetration and is very suitable for high-resolution deep imaging of living tissues (156). The idea of multiphoton excitation was first proposed by Maria Göppert-Mayer et al., and Franken et al. conducted related works in nonlinear optics (157, 158). The principle of TPM is that when two or more photons of a higher wavelength hit the fluorophore simultaneously, they are absorbed, resulting in fluorophore excitation and emission of light at half wavelength (**Figure 1D**). In 1963, Kaiser et al. reported the first two-photon excitation of CaF_2_:Eu^2+^ fluorescence (159). Later, Denk et al. achieved two-photon fluorescence microscopy by a scanning microscope with ultrafast pulsed lasers (160).

TPM is an alternative to conventional single-photon confocal microscopy. Compared with single-photon confocal microscopy, TPM has three advantages. The first advantage is its ability of deep-tissue imaging (161, 162); TPM uses longer wavelengths which are less subject to absorption and scattering effects compared to the shorter wavelength light used in single-photon confocal imaging (162). TPM also exhibits increased efficiency compared to single photon confocal imaging by collecting all useful information about a single location to generate images (162, 163). This advantage also contributes to its high-resolution imaging in deep tissues, because higher fluorescence collection efficiency means greater signal intensity (higher photon flux) (164). Finally, photobleaching and photodamage are limited to narrow region around the focus by TPM (160, 165).

To date, TPM has been widely used in cellular and subcellular imaging in various organs of living animals (166, 167). TPM is a promising technique for retinal imaging given its ability to detect both structural and biochemical processes of the eye. In 2004, Imanishi et al. first applied TPM to the eye, revealing previously uncharacterized structures (retinosomes) distinct from other cellular organelles in the dissected mouse eye (168). Maeda et al. used TPM to characterize the early phase of retinal degeneration (169). Palczewska et al. developed an AO-TPM system to monitor early molecular changes in retinoid metabolism related to eye diseases (141).

The research on eye detection by TPM is incomplete and limited to animal models due to its safety concerns in human use with nonlinear optical effect (170). Nevertheless, it is still a promising tool for human disease detection, considering the absorption spectra of human eye tissues and the biochemical process revealed by TPM (171–174). Another key advantage of TPM is that it offers the possibility of imaging intrinsic fluorophores that are outside the normal transmission window of the eye. Palczewska et al. found that reducing the pulse repetition frequency and lowering the average laser power is a viable option to improve safety (141). Alternatively, the use of infrared lasers at the maximum permissible exposure ensures both corneal and retinal protection, all while maintaining imaging efficacy (175). However, work is needed to make this technology suitable for clinical utilization. Future studies may improve TPM acquisition techniques by exploring safety problems in animal models and balancing high-resolution imaging with the reduction of the laser power (176–178).

### 2.5 Light-sheet fluorescence microscopy

Light-sheet fluorescence microscopy (LSFM) is a fluorescence microscopy with good optical sectioning capability (**Figure 1E**). In 1903, Siedentopf and Zsigmondy described the first version of LSFM (ultramicroscopy) (179). They projected sunlight through a split aperture to directly observe gold nanoparticles. Many years later, researchers rediscovered the idea of using light-sheets to image, after which, many methods like orthogonal-plane fluorescence optical sectioning (OPFOS), selective plane illumination microscopy (SPIM), and digitally scanned laser light-sheet microscopy (DSLM) were reported (180–182). The milestone of LSFM was in 2004 when Huisken et al. developed SPIM to generate multidimensional images of live embryos with high resolution (181). This breakthrough accelerated the rapid development of LSFM (183). In a conventional LSFM, the sample is illuminated by a thin laser beam (a sheet of light). The fluorescence from the illuminated plane is collected in a perpendicular direction from the light-sheet axis. Therefore, the light can be captured by a camera and be generated into an image (184). Because the sample is only illuminated by a thin sheet of perpendicular light (2–6 µm), LSFM has the advantage of reduced photodamage/bleaching over other fluorescence microscopic techniques, such as confocal fluorescence microscopy and TPM (185, 186). Accordingly, LSFM can achieve high-speed and high-resolution imaging while using relatively less light energy. Additionally, new advancements have integrated AO with LSFM, addressing issues relating to image degradation caused by sample-induced optical aberrations (187, 188). More recently, LSFM has been applied in retinal imaging. Luo et al. demonstrated the applicability of LSFM for the assessment of optic nerve regeneration by imaging the optic nerve and brain of the mouse (189). Icha et al. used the multi-view fusion method to image the developing zebrafish eye (190). Prahst et al. illustrated the potential of quantitative 3D/4D LSFM imaging in understanding human eye especially pathological, neuro-vascular, and degenerative processes (191). Current LSFM techniques require sample preparation including hydrogel embedding and hooks (186); therefore, it still cannot be used in imaging live animal eyes and human eyes.

### 2.6 Limitations and clinical challenges of cellular-resolution retinal imaging

To facilitate comparison across modalities, **Table 1** summarizes the major technical and practical characteristics of OCT, AO-based imaging, TPM, and LSFM from a clinical perspective. Each modality also faces important limitations that must be considered when interpreting results or envisioning future clinical translation. Although OCT is widely used clinically (199), achieving reliable cellular-resolution imaging remains challenging. Raster scanning introduces motion artifacts, especially during B-scan stacking for volumetric imaging. In vis-OCT, shorter wavelengths improve axial resolution but decrease penetration depth and impose stricter light safety limitations, potentially requiring reduced field of view or slower acquisition. AO enhances lateral resolution but adds substantial system complexity, including wavefront sensing, deformable mirrors, and demanding optical alignment (200). These systems remain sensitive to fixation instability, tear-film fluctuations, and small eye movements, all of which can degrade AO correction (201). TPM provides powerful functional and fluorescent imaging capabilities but is fundamentally constrained by light safety: nonlinear excitation requires high peak power that exceeds safe exposure limits for the human retina. Tissue scattering further limits penetration depth, confining TPM imaging largely to superficial structures in rodent eyes. Many TPM applications also require exogenous fluorescent labeling, which precludes human use. LSFM enables rapid, volumetric, high-contrast imaging of ex vivo tissue, but its geometry—requiring orthogonal illumination and sample mounting—precludes *in vivo* ocular application. It is also incompatible with natural eye motion, and thus currently serves exclusively as a tool for studying retinal organization, vascular architecture, and developmental processes in fixed or cleared specimens.

**Table 1:** Specifications and features of *in vivo* cellular-resolution imaging modalities.

Image modality	Image type	Recording	Species	Resolution		Penetration (Retina)	Clinical potential	Validated structures	Unique advantages	Key limitations
				*Ax*	*Lat*					
Optical Coherence Tomography (OCT) (192)	volume	Raster scanning	Human, mouse	2-5 µm	~15 µm	Full retinal thickness	Widely available (clinical standard)	All retinal layers (lamination)	Noninvasive, fast volumetric imaging, lamination visualization	Lateral resolution insufficient for cellular imaging
Visible-Light OCT (vis-OCT) (193)	volume	Raster scanning	Rat, mouse	1-2 µm	4-10 µm	Full retina; reduced penetration to RPE–choroid	Experimental, phototoxicity concerns	INL somas, RGC axon bundles, outer segment reflectivity	High axial resolution, oximetry, enhanced layer contrast	Light safety limits, motion sensitivity
Adaptive Optics SLO (AO-SLO) (140, 194)	en face/volume	Point-scanning	Human, primate	NA	~2.5 µm	Outer retina (PRs), limited inner retina	Limited to research centers	Cone spacing, rod structure (split-detection), RPE autofluorescence	Cone/rod mosaics, RPE autofluorescence, microglia dynamics	Small FOV, long acquisition time, technically complex
Adaptive Optics OCT (AO-OCT) (195)	volume	Raster scanning	Human, mouse	~3 µm	~3 µm	Full retina	Research only	RGC somas, RPE mosaic, photoreceptors	3D cellular imaging (PRs, RGC somas, RPE mosaic)	Motion sensitivity, high cost, complex alignment
Two-Photon Microscopy (TPM) (196)	volume	Point-scanning	Mouse	~10 µm	~2 µm	Inner retina only in rodents	Not clinically feasible, requires high peak power	Photoreceptor retinoids, microglia, bipolar terminals	Visual cycle imaging, fluorescence, cellular signaling	Unsafe for humans; limited penetration; requires dyes/genetic labels
Adaptive Optics TPM (AO-TPM)) (197)	volume	Point-scanning	Mouse	~9 µm	~1 µm	Slightly improved; still limited to rodents	Research only, same safety constraints as TPM	RGC somas, dendritic arbors, microglia dynamics	Subcellular resolution, microglia/RGC dynamics	Not translatable to clinic
Light Sheet Fluorescence Microscopy (198)	volume	Snapshot/parallel plane	Mouse, zebrafish	~25 µm	~3 µm	Thick tissue (but ex vivo	Not for *in vivo* human or animal eye	Microvasculature, neuronal lamination, optic nerve regeneration	Large-volume 3D/4D imaging, vascular reconstruction	Requires enucleation or cleared tissue; not applicable *in vivo*

Despite differing optical architectures, several challenges are shared across all high-resolution retinal imaging techniques. Motion artifacts from microsaccades, respiration, and cardiac pulsation impair the stability needed for cellular visualization. Light safety constraints limit photon flux, especially in vis-OCT and nonlinear microscopy. Small fields of view prolong acquisition time and complicate clinical workflows. Furthermore, large data volumes require advanced registration, denoising, and segmentation algorithms to achieve clinically usable outputs. Partial histological validation for some modalities (e.g., vis-OCT, AO-OCT) means that certain cellular features remain interpretive rather than definitively confirmed. Additionally, cost, complexity, and lack of commercial systems continue to hinder translation of AO and ultrahigh-resolution OCT into everyday practice. Advances in ultrafast imaging (MHz OCT (202), line-scanning scheme (203), computational aberration correction, more efficient light sources, real-time motion tracking, and machine-learning-based denoising and segmentation are expected to reduce many of these barriers. Continued integration of *in vivo* imaging with histological validation and longitudinal functional studies will also expand clinical confidence and interpretation of cellular-scale biomarkers.

## 3 Applications in imaging retinal cells

The organization of this section follows the flow of visual information through the retina. We begin with photoreceptors, which transduce light into electrical signals, then proceed to bipolar and amacrine cells in the inner nuclear layer, and finally to retinal ganglion cells, the output neurons of the eye. After covering the neuronal visual pathway, we review the supporting cell types essential for retinal homeostasis and disease processes, including the retinal pigment epithelium (RPE), immune cells such as leukocytes, microglia, and Müller glia. This structure emphasizes the functional circuitry of vision rather than strict anatomical layering, while still covering all major retinal cell classes.

### 3.1 Photoreceptor cells

**Figure 2:**
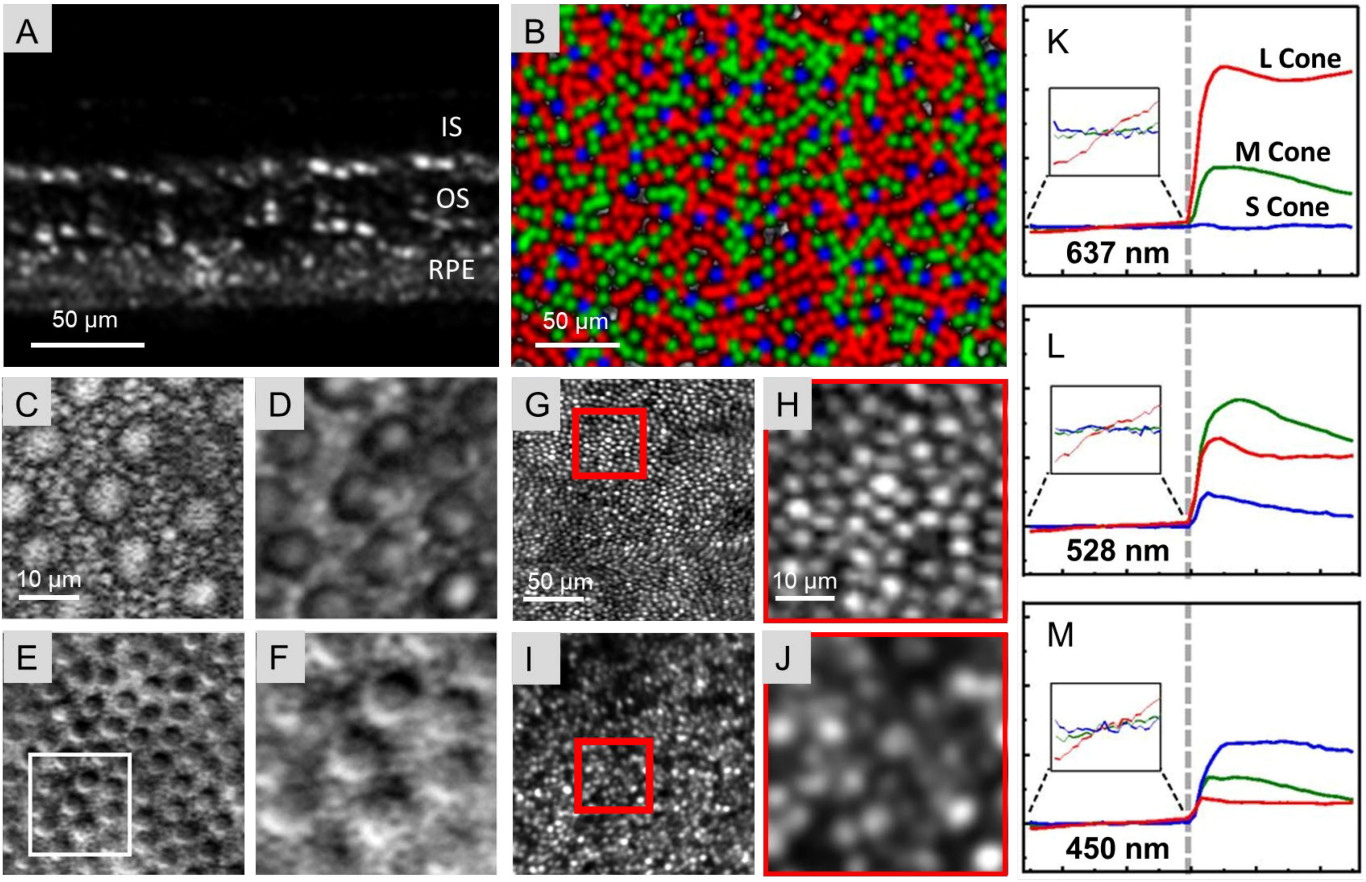
*In vivo* images of photoreceptors shown through different image modalities. **(A)** AO-OCT image of the human retina measured at 5 degrees temporal to the fovea; approximately 10 registered B-scans were compiled for this image. (Reproduced/adapted from Wells-Gray et al., 2018). **(B)** Color-coded map of the trichromatic cone mosaic in a human subject (small = blue; medium = green; large = red). (Reproduced/adapted from Zhang et al., 2019). **(C-F)** Offset-aperture images showing cones from monkeys through multiple imaging modalities in a 40 × 40 µm field of view. Cones are seen in each imaging modality while the visibility of rod photoreceptors varies. The white square in E depicts the location of zoomed-in views **(C, D, F)**; C = Two-Photon Excitation Fluorescence; D = Confocal; F = multi-offset. (Adapted from Rossi et al., 2017). **(G-J)** AO-OCT *en face* images of the cone mosaic (G+I) and their zoomed in images (H+J) in a healthy human control (G+H) and a patient with Retinitis Pigmentosa (I+J). (Adapted from Lassoued et al., 2021). **(K-M)**: Phase response of cones varies with cone type (S, M, L) and wavelength of the stimulus. Major tick marks on the x-axis represent time in seconds. The dashed gray line at 0 seconds represents the onset of the 5-ms stimulus flash. The phase response was referenced to the average of the prestimulus optical path length. Cone responses are colored red (L), green (M), or blue (S) based on the k-mean classification and expected spectral sensitivity of each cone type to the stimulus wavelength. Average responses of the grouped traces are shown in graphs **(K-M)**. (Adapted from Zhang et al., 2019).

Photoreceptor cells are a primary interest of cellular resolution retinal imaging given their role in the visual processing circuit and various retinal diseases. Located in the outer retina (**Figure 2A**), these cells can be visualized and quantified *in vivo* by a variety of imaging modalities. The application of AO to SLO and OCT technologies has greatly facilitated the ability to achieve cellular resolution imaging of these cells in both animals and humans (**Figure 2**). (2, 63, 204–209) Several existing papers summarize the related studies on photoreceptors and limitations of AO-SLO (59, 63, 210–212) and AO-OCT (206, 207, 213) imaging. Here, we will focus on the emerging techniques and studies for visualizing and quantifying photoreceptor appearance and their functional response.

Cones can be confidently visualized using confocal AO-SLO and there are already couple of specific review papers (63). However, rods are difficult to image with AO-SLO techniques given their relatively smaller size and variable reflectance. Recently, Scoles et al. used non-confocal split-detection AO-SLO to view peripheral cone and rod inner segments in humans, the location of which can be correlated to the cones seen ex vivo in confocal images of donor eyes (214) (63). Accordingly, split detection, image averaging, as well as decreases in pinhole size and imaging wavelength have been proposed to improve rod visualization (63, 215). Significantly, Tan et al. noninvasively captured nanometer-scale, light-evoked deformations not only in cones but also in rods, the RPE, and the subretinal space (216). This method allows for detection of scotopic rod responses down to 0.01% bleach levels with functional mapping across a 12°field in a single flash, making it incredibly useful in detecting early rod disfunction and monitoring disease progression (216).

Notably, Rossi et al. explored the visualization of photoreceptor cells and other retinal cells using an non-confocal offset-aperture detection scheme of AO-SLO in monkeys and humans (**Figures 2C-F**) (2). Recently, vis-OCT (71) without AO has been explored to observe photoreceptor cell somas in the outer nuclear layer due to the high resolution and strong scattering in visible light band; yet, in comparison, current vis-OCT systems are less effective than the IR AO-OCT systems. In addition to these approaches, a recent multi-MHz phase-stable SS-OCT developed by Lee et al. demonstrated 3D, depth-invariant cellular-resolution imaging of the living human retina over a 3 × 3 mm field, presenting a significant improvement in field of view at the given resolution (217). Additionally, detection and quantification of photoreceptor functional response to light has also been of great interest in recent works, as the ability to do so with non-invasive methods holds great promise in both disease diagnosis (**Figures 2G-J**) and treatment (218–222). These efforts were focused on measuring the length of individual cone cells (19). Gofas-Salas et al. discussed using non-confocal split-detection AO-SLO to visualize inner photoreceptor segments, even if the outer photoreceptor segments have been damaged in pathological or methodological processes (223–225).

Optoretinograms (ORG) are an exciting tool in this field, given their ability to provide measurements of retinal structure and functional cellular response (218, 221, 226).While traditional methods of microperimetry and electroretinography (ERG) provide aggregate assessments of photoreceptor function, they cannot provide results at the individual cellular level (218). Again, AO holds great promise in this area, providing cellular resolution imaging of light-induced photoreceptor functional changes in the intact human eye (218). ORG has been categorized into intensity-based and phase-based subtypes (218). Given that previous studies combining AO-OCT-SLO technologies have been used to differentiate between rods and cones and visualize the processes within each cell type (19, 227), these modalities are particularly applicable to this area. Simultaneous AO-OCT and AO-SLO imaging systems have been measured photoreceptor responses to light stimuli (215). Accordingly, intensity-based protocols have demonstrated that different types of cones respond differently to light stimulus intensity and wavelength (**Figures 2K-M**) (208, 218, 219) Bernucci et al. recently utilized a supercontinuum laser-based, AO-OCT ORG to objectively measure cone sensitivities across the visible light spectrum (228). Such systems have separately been used to document the transient deformation that photoreceptor outer segments undergo as a result of light exposure and phototransduction (220, 229), which was later validated with histological examination and ERG (230). Given the physical deformations that occur during cell function, photoreceptor outer segments have been described by some as particularly suited for ORG technology (231). Phase resolved AO-OCT is able to capture images with sufficient speed and resolution to track changes in the optical path lengths of outer segments (219). Pandiayan et al. introduced a 16 kHz line scan AO-SD-OCT system with an anamorphic detection paradigm which improved roll-off and light capture efficiency. This system can also be tailored to the relevant clinical application, capturing areas from an individual photoreceptor to 100um wide (219). Phase-sensitive AO-OCT systems have been applied to the retinitis pigmentosa disease model, allowing for detailed cellular-level tracking of functional responses in cone cells (222).

### 3.2 INL cells: bipolar and amacrine cells

**Figure 3:**
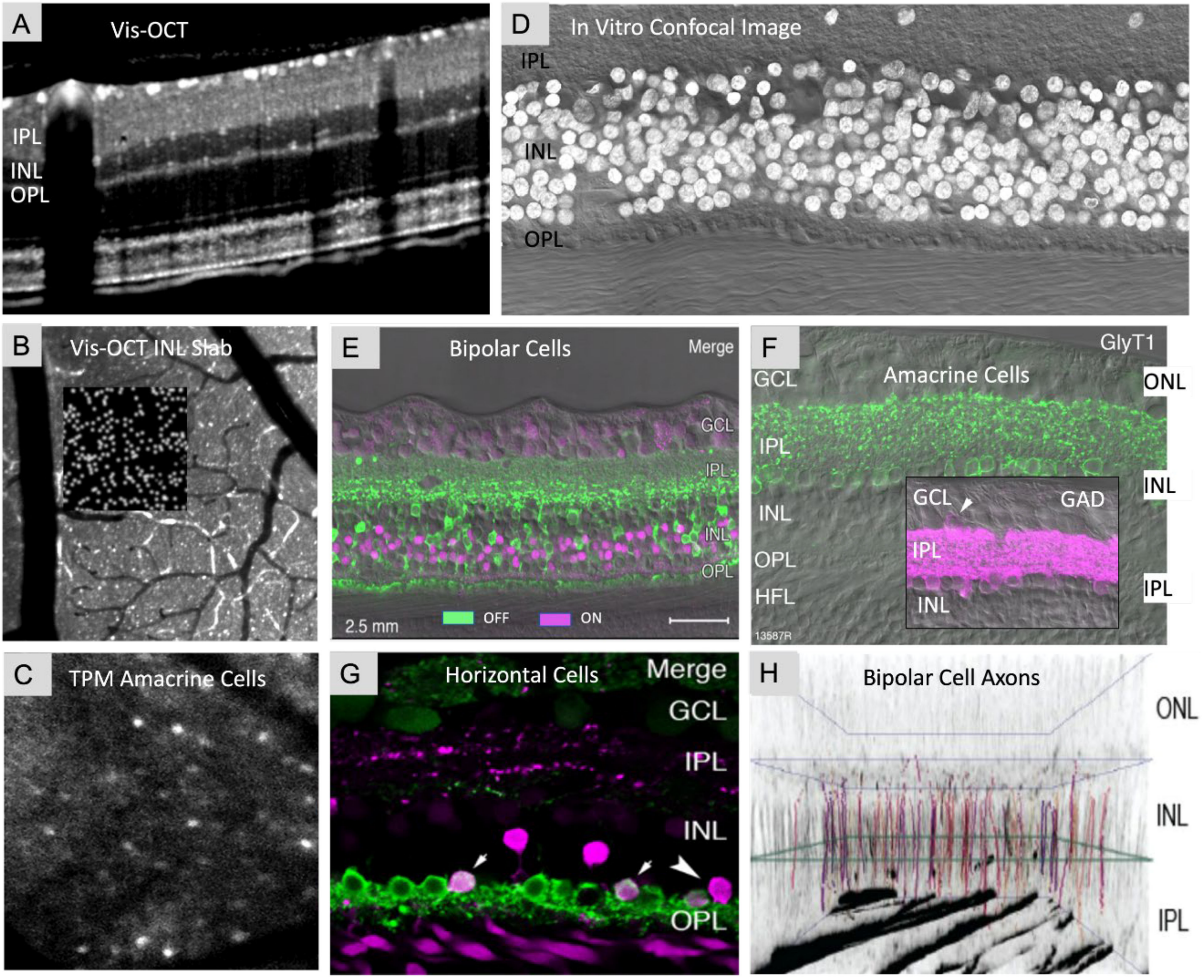
INL cell types revealed in different imaging modalities. **(A)** Cross-sectional image of rat retina acquired *in vivo* using vis-OCT shows the cells in INL layers. (From Pi et al., 2020). **(B)***En face* projection of INL slab in vis-OCT images delineates INL cell bodies. (From Pi et al., 2020). **(C)** Amacrine cells in mouse retina is labeled by injecting AAV-EF1α-FLEX-Twitch2b into VGat-Cre transgenic mice and imaged *in vivo* with two-photon microscopy (TPM). (From Wang et al., 2021). **(D)** Confocal image of a vertical section through a postmortem human donor retina (age 36 years) visualized INL cell bodies labeled with DAPI nuclear staining. (From Masri et al., 2021). **(E)** Confocal image revealed the OFF-midget (labeled with antibodies against recoverin, Green) and ON (labeled with antibodies against islet-1, Magenta) bipolar cells. (From Masri et al., 2021). **(F)** Confocal image of amacrine cells in the human retina labeled with antibodies against glycine transporter 1 (GlyT1, green) for glycinergic amacrine cells, or glutamic acid decarboxylase (GAD-6, magenta) for GABAergic amacrine cells. Displaced GABAergic amacrine cells are visible in the ganglion cells layer (arrowhead) but were not quantified. (From Masri et al., 2021). **(G)** Confocal image revealed the horizontal cells in the human retina processed with antibodies against parvalbumin (green) and calbindin (magenta) and shows that H1 horizontal cells expressing parvalbumin alone and H2 cells expressing both parvalbumin and calbindin (arrows). A cell body expressing calbindin alone can also be observed (arrowhead). (From Masri et al., 2021) **(H)** Bipolar cell axons were identified and traced with second harmonic generation (SHG) imaging in a transgenic green fluorescent protein (GFP) mouse. Note that terminal of three subtypes of bipolar cells (CBC4: type 4 cone bipolar cell, CBC7: type 7 cone bipolar cells, RBC: rod bipolar cells) are distinguishable and penetrate to different depth in IPL by the overlap with GFP at zero IPL (green square). (From Arafat Meah, et al., 2022].

A variety of imaging modalities have been used to image this cell layer in animal models. Rare studies have demonstrated that AO technology is capable of providing high resolution images of bipolar cells in live human eyes (232). More recently, cells within the INL have also been observed indirectly. Liu et al. utilized AO-OCT combined with long acquisition times to observe moving organelles and, thus, infer RGC soma locations (233). The images obtained from this study demonstrated the RGC dendrites and synapses with bipolar and amacrine cells within the INL (233). RGC soma projections were then spatially mapped to infer the locations of underlying INL cells, photoreceptors and RPE cells with through focus-imaging (233). Given its ability to resolve cells at multiple retinal depths and define reflectance of cell somas in visible light range, vis-OCT may be uniquely positioned to observe INL cells. Pi et al. recently reported using vis-OCT volumetric registered and averaged images to view cells in the inner nuclear layer of rats (**Figures 3A, B**) (71). Previously, Zhang et al. utilized temporal speckle-averaging of infrared OCT images to visualize INL cells (234). Comparably, vis-OCT resulted in more detailed imaged of INL cell bodies, along with RNFL bundles and photoreceptors (71). Schroeter et al. used two-photon microscopy and time-lapse confocal microscopy to image retinal bipolar cell axon terminals in live zebrafish (235). Lu et al. previously used adeno-associated viruses to transduce specific promoters and enhancers in ON-type rod bipolar cells in both mice and marmoset monkeys (236). Similarly, Wang et al. utilized transpupillary two-photon fluorescent imaging to provide cellular resolution of amacrine cells in mice retinas *in vivo* (**Figure 3C**) (237). Recent advancements improved on two-photon fluorescent imaging with AO, enabling clear visualization of synaptic structures and dynamic retinal changes in disease models for mice (68). Using a similar method, functional calcium imaging of INL neurons was also achieved (197). These authors used VGAT-Cre transgenic mice injected with an adeno-associated virus vector to encode a fluorescence-based calcium sensor with yellow fluorescent proteins (237). By adapting a standard multiphoton microscope, the optical set up described by these authors provides a relatively simple approach to both short- and long-term experiment durations with minimal animal stress (237). In addition to structural imaging, ORG offers promise for future wide-field, layer specific structural and functional imaging of INL cells due to the method’s high sensitivity and axial resolution (216, 238).

However, reports of *in vivo* visualization of retinal bipolar cells are still limited with histology remaining as the standard approach to visualize the INL cells. Fluorescent techniques have been used to directly observe INL cells in postmortem human retinal samples (**Figure 3D**) (239); specifically, they have revealed antibody-labelled OFF- and ON-Bipolar cells (**Figure 3E**) (239), amacrine cells (**Figure 3F**) (239), and horizontal cells (**Figure 3G**) (240). Such techniques highlight the spatial orientation between INL cell types (239). Studies employing transgenic green fluorescent protein mice and second harmonic generation imaging revealed that the axons of different bipolar cell types extend to varying retinal depths (**Figure 3H**) (240).

### 3.3 Retinal ganglion cells

#### 3.3.1 RGC somas

RGCs share many features with neurons in the CNS and possess similarities to the brain in terms of anatomy, function, as well as immunologic and insult response (1). They are transparent and thus less contrast for optical imaging. Applications of AO has perhaps been the most successful in improving contrast imaging of RGCs and visualizing individual ganglion cells *in vivo* (63, 233). Previous imaging of inner retinal cells with AO-SLO has required transgenic models, induced fluorescent protein expression, or administration of exogenous contrast (63). For instance, fluorescent confocal AO-SLO has been utilized to visualize the somas, dendrite processes, and axons of labeled ganglion cells in the mouse retina *in vivo* (205, 241). Two-photon fluorescence AO-SLO has also been used to visualize these structures, allowing for the identification of various cellular features depending on the depth of focus for the incident beam (242). These authors were able to perform 5-µm axial sectioning and resolve dendrites in different layers (242).

**Figure 4:**
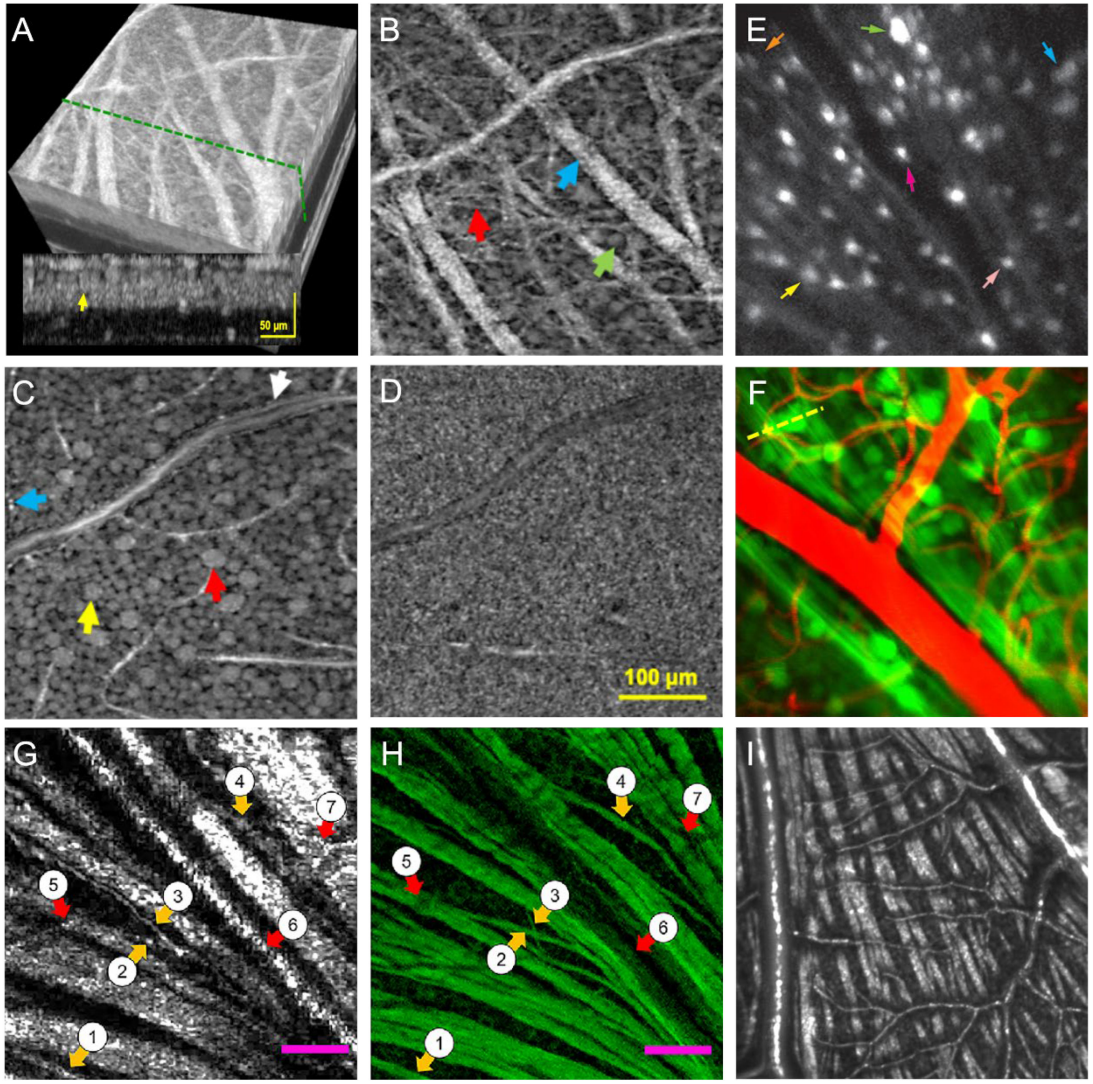
Images of RGC somas. **(A)** Three-dimensional registered and averaged AO-OCT volume with green dashed line signifying the cross-sectional B-scan shown in the inlay. Yellow arrow within inlay indicates the same GCL soma seen in **(C)** Images shown in B-D were extracted at depths of 13, 22, and 46 μm below the inner limiting membrane (ILM). Scale bar in D also applies to B and **(C, B)** A complex network of nerve fiber bundles of varying sizes ranging from 30 μm (blue arrow) to 3 μm. GCL somas are seen near the image bottom (green arrow). **(C)** GCL somas of varying sizes, as indicated by the color arrows. The red arrow points to a large soma, while the blue and white arrows depict edges of vessel walls and the close proximity of GCL somas. **(D)** Dense connections between bipolar synapses and dendrites of ganglion and amacrine cells. (Images A-D and corresponding caption adapted permission from Liu et al., 2017). **(E)** Vglut2-Cre transgenic mice labelled by injecting AAV-EF1α-FLEX-Twitch2b were used to image RGCs with *in vivo* two-photon techniques. (Reproduced from Wang et al., 2021). **(F)** Full AO-TPM images depicting RGCs (green) and blood vessels (red). (From Qin et al., 2020). **(G, H)** Magnified images comparing *in vivo* vis-OCT fibergraphy **(G)** and *ex vivo* confocal microscopy **(H)** images of RGC axon bundles. Orange arrows 1–4 signify small RGC axon bundles visible in both imaging modalities. Red arrows 5–7 demonstrate a comparison of blood vessels. Scale bars: 50 µm. (Adapted with permission from Miller et al., 2020). **(I)***En face* vis-OCT image demonstrating nerve fiber bundles.

Rossi et al. was the first to utilize confocal AO-SLO to image individual neuron somas in the RGC layer of monkeys and humans without fluorescent labels or high light levels (2). Concurrently, Liu et al. described an AO-OCT technique with highly-processed singly-scattered light to image RGC somas *in vivo* in the human retina (**Figures 4A-D**) (233), through long acquisition times and 3D subcellular image registration and using moving organelles functioned as a contrast agent for the inference of soma locations (233).

Recent advancements have applied machine learning to further enhance RGC imaging using AO-OCT. Two models — RGC-CCT and RGC-CPS — leverage both labeled and unlabeled datasets to identify RGC somas in 3D AO-OCT volumes, outperforming traditional fully supervised methods (150). Further, Zhang et al. used temporal speckle averaging (TSA) of AO-OCT and OCTA images to increase image contrast and reduce signal to noise ratio; this method provided cellular-resolution images in mice eyes without extrinsic contrast agents, relying on temporal evaluation of speckle patterns to reduce noise via image stacking (234). Gofas-Salas et al. recently demonstrated a radial multi-offset detection AO-SLO can be used to image RGCs; in this system, a single wavelength source afforded increased power while decreasing optical aberrations (223). AO-SLO combined with calcium imaging has been performed *in vivo* in macaques to measure the RGC response to light (243). Additionally, calcium response measurements in RGCs have been used to visualize the efficacy of vision restoration techniques; Cheong and colleagues utilized a calcium indicator to confirm uptake and expression of a viral vector in RGCs from retinal degeneration mouse models (244).

Later, Laforest et al. described a transscleral illumination approach in humans in which RPE and choroid backscattered light is used to illuminate the inner retinal layers, which are then imaged through a non-dilated pupil to produce dark field images (245). When combined, this technique generates phase imaging with double the pupil’s numerical aperture (245). While the technique described by Liu and colleagues required relatively long capture durations (10 minutes), the technique of Laforest et al. demonstrated rapid retinal imaging which would be more conducive to clinical translation, particularly in subjects who have difficulty with target fixation (233, 245). Continual improvements in the applications OCT technology have also improved imaging of these cells. Pfäffle et al. utilized phase evaluation algorithms to full-field SS-OCT data to demonstrate GCL and IPL activation in human eyes *in vivo* (246). Notably, signals from these layers were only visualized after post-imaging processing suppressed artifacts induced by blood pulsations and motion (246). Visible-light OCT combined with volumetric registration and image averaging has been also used to observe RGC somas within the ganglion cell layer (71), while combination OCT and confocal SLO systems have been used to obtain longitudinal images of single RGCs (247).

Standalone TPM methods and those using AO have also been described. Wang et al. visualized RGC somas and axon fascicles *in vivo* using Vglut2-Cre transgenic mice injected with an adeno-associated virus vector to encode a fluorescence-based calcium sensor with cyan fluorescent proteins (**Figure 4E**) (237). Qin et al. used an AO-TPM system with a nonlinear fluorescent guide star to obtain subcellular (submicron) resolution for *in vivo* mice retinas (**Figure 4F**) (197). Importantly, these authors combined ocular aberration correction with a diffraction-limited point spread function to maximize efficiency of the photon excitation, revealed neuron soma and dendrite images (197).

#### 3.3.2 Nerve fiber layer (RGC axons)

AO-SLO imaging, and in combination with other technologies, has been used to image RNFL and correlate these findings to biomarkers of human disease (248–250). Geng et al. utilized fluorescent confocal AO-SLO to directly visualize nerve bundles in transgenic mice *in vivo* (205).TPM imaging has also proved useful in RNFL imaging. Jayabalan et al. obtained real-time, concurrent two-photon ICGA and FA images using a single light source; in this system, two-photon FA was used to image the RNFL of rabbits and rats (251). These authors reported that efficiency of the two-photon technique can be optimized through the modification of pulse duration and excitation wavelength; this system can also be modified to image multiple animal models (251). Single light source systems have also been described in AO-OCT. Jian et al. described an AO-FD-OCT setup with a one light source for both wavefront detection and image capture (252). This system utilized a refraction cancelling lens to decrease corneal back reflection and lower-order visual aberrations, resulting in increased contrast and brightness of nerve fiber bundles in mice retinas *in vivo (*252*).* These authors later combined wavefront sensorless AO with FD-OCT, which allowed for precise depth selection and focus on specific retinal structures (61).

More recently, Miller at al. visualized individual RGC axon bundles of varying sizes in mice using visible-light OCT fibergraphy (**Figures 4G, H**) (253). These authors quantitatively compared the results obtained through vis-OCT imaging with the confocal microscopy of flat-mounted, antibody-labelled specimens, finding agreement between imaging results and demonstrating the potential for vis-OCT as a non-invasive method for visualizing these axons (253). Further, visible-light OCT combined with volumetric registration and image averaging has been used to image RNFL bundles in rat eyes (**Figure 4I**) (71). The resulting images were roughly equivalent to those AO-OCT when imaging for RNFL bundles; however, vis-OCT was able to capture a wider field of view (71). Notably, speckle noise remained an issue that affected image quality in vis-OCT images. Elsewhere, scan modulation has been used in vis-OCT systems to improve image quality by increasing the contrast-to-noise ratio in *in vivo* images by 2.35 dB (254). Future applications of vis-OCT technology with advanced image processing may decrease the number of images needed to average to obtain adequate resolution, while minimizing the transient photoreceptor bleaching that occurs following visible light exposure (71).

### 3.4 Retinal pigment epithelium cells

**Figure 5:**
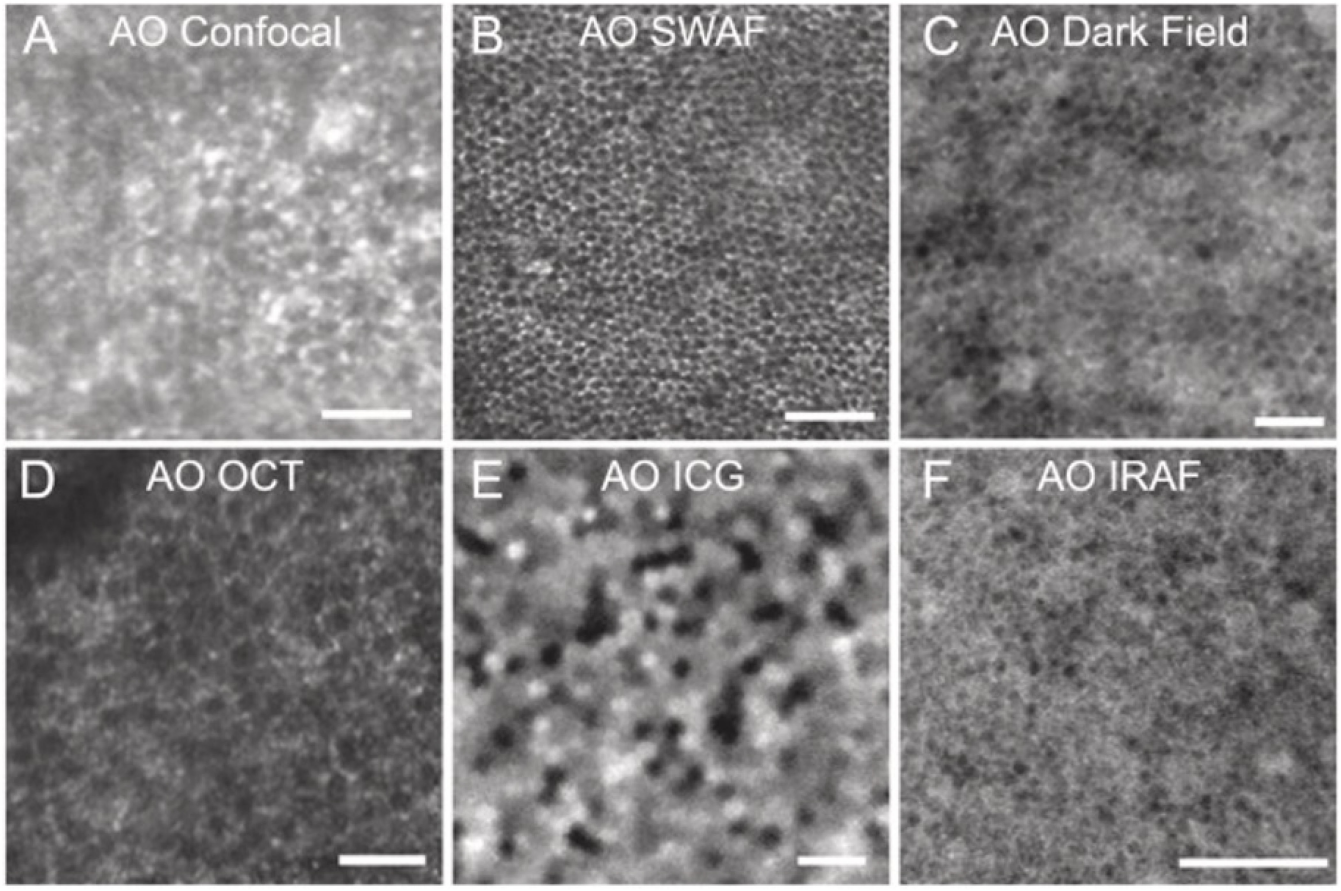
Retinal pigment epithelial (RPE) cell mosaics shown through various imaging modalities. **(A)** Confocal AO-SLO images of RPE cells from a patient with cone-rod dystrophy (Reprinted from Roorda et al., 2007). **(B)** AO-SLO images augmented with short wave autofluorescence demonstrate RPE cells in a monkey (Reprinted from Morgan et al., 2009). **(C)** Dark-field AO-SLO images of RPE cells from a healthy human subject (Reprinted from Scoles et al., 2013). **(D)** AO-OCT images of human RPE cells (Reprinted from Liu et al., 2016). **(E)** AO-ICG visualization of human RPE cells (Reprinted from Tam et al., 2016). **(F)** Human RPE cells shown under AO infrared autofluorescence (Reprinted from Liu et al., 2017). Scale bars, 50 mm. Figure and caption adapted with permission from Wynne et al.,2021.

Although anatomically the RPE lies externally to photoreceptors, we present it after the neuronal pathway because of its role as a metabolic and structural support system for all photoreceptor-dependent visual processing. A variety of modalities have been employed to image RPE cells, including a variety of AO and fluorescence-based imaging (63, 255–260). In a recent review, Wynne et al. discussed the opportunities and challenges in imaging RPE cells owing to their low internal contrast and high light scattering properties (63). Fundus autofluorescence (FAF) has been frequently used to image RPE cells due to its intrinsic fluorophores, lipofuscin and melanin (63). A recent review by Schmitz-Valckenberg and colleagues describes the utility of FAF, as well as the variety of fluorophores that can used in both animal models and humans (261). Roorda et al. first used AO-SLO to image the human RPE layer in patients with cone-rod dystrophy, finding that the RPE was more easily visualized without the presence of photoreceptors (**Figure 5A**) (255). Morgan et al. used AO-SLO to the detect autofluorescence of the RPE cells in live monkeys with healthy photoreceptors (256). The concentration of cytoplasmic lipofuscin was thought to contribute to the autofluorescence pattern seen in **Figure 5B** (63, 256). Since this work, recent studies have evaluated melanosome and lipofuscin granule density in the RPE cells of transgenic mice *in vivo* using small wavelength autofluorescence (SWAF) with directional backscattering (262). The same study also utilized directional OCT and spectrometrically-integrated SLO to evaluate RPE melanolipofuscin and lipofuscin granule density (262). Transscleral optical phase imaging (TOPI) has also been used to obtain high resolution depictions of all retinal layers, including the RPE. By applying near-IR light transsclerally, the cone reflectivity that accompanies trans-pupil illumination is avoided, resulting in high contrast structural images of the RPE (63, 263).

Lipofuscin visibility on dark-field AO-SLO has been used to image RPE cells at certain eccentricities with low light levels and lessened subject discomfort, but sacrificing contrast and image quality(**Figure 5C**) (63, 257). More recently, Jayabalan et al. described TPM imaging as an efficient way to detect minute changes in lipofuscin distribution, alongside functional and structural retinal information (251). These authors discussed TPM imaging in comparison to confocal FAF, which provides relatively lower resolution estimations of lipofuscin concentrations (251). Accordingly, confocal fundus autofluorescence could only detect RPE changes after significant decreases in lipofuscin concentrations (251), potentially making TPM a more sensitive methodology for future studies. Interestingly, AO-two photon techniques have been used in primates, but at light levels that would be phototoxic in humans (264).

Additional adaptations of AO technology have been used to visualize the RPE with subcellular resolution. Liu et al. utilized organelle motility as a contrast medium to view human RPE cells *in vivo* with AO-OCT (**Figure 5D**) (265). Through image averaging, these authors were able to increase contrast and produced three-dimensional reflectance profiles of the RPE mosaic. This group later combined AO-OCT with speckle field dynamics to quantify organelle dynamics, as an evaluation of cellular health and functionality (198). Recently, an AI model (P-GAN) has been developed to clean up speckle-obscured RPE cellular features from a single AO-OCT volume, eliminating the need to capture and average multiple scans, thus reducing imaging time (151). Using P-GAN, Das et al., were also successful in providing improved RPE cell contrast by 3.5-fold (151). In future studies, organelle motility may be an important biomarker in visualizing overall RPE health status (198, 265).

In addition to the studies using endogenous fluorophores, exogenous dye sources have also been explored. Indocyanine green (ICG) dye heterogeneously localizes to the RPE layer after systemic injection (260) and remains stable for up to 24 hours (63). Tam et al. confirmed that AO-ICG ophthalmoscopy is able to image individual RPE cells within the RPE mosaic in living human eyes (**Figure 5E**) (260). Liu et al. combined AO with infrared-autofluorescence to measure the density of the RPE mosaic in healthy human eyes (**Figure 5F**) (233). With this modality these authors were able to calculate the number of cone cells supported by each RPE cell. Grainger et al. used short-wavelength autofluorescence (SWAF) and infrared autofluorescence (IRAF) to characterize *in vivo* morphometry and multispectral autofluorescence of the retinal pigment epithelial (RPE) cell mosaic and its relationship to cone cell topography across the macula. Future applications of these techniques may improve the understanding of the many disease pathologies influenced by RPE cell dysfunction or destruction.

### 3.5 Immune cells

#### 3.5.1 Leukocytes

Label-free recording of immune cell activity is particularly important, as it is unknown if exogenous labels alter biologic immune response in the tissue microenvironment. Joseph et al. imaged myeloid cell dynamics in live mouse retinas using label-free phase contrast AO-SLO and time-lapse videography (15). These authors induced uveitic conditions by injecting lipopolysaccharide into the eye and subsequently measured cell motility during acute inflammatory reactions from their onset to resolution. They were able to observe leukocyte tissue infiltration, including rolling, crawling, and trans-endothelial migration stages, as well as differentiate tissue resident retinal neutrophils (15). Notably, their methodology utilized near-infrared light (796nm) at lower power levels than required in multi-photon techniques, thereby minimizing the risk of phototoxicity (15). In a subsequent experimental stage, these authors used fluorescent antibody labeling to confirm neutrophil location; interestingly, they reported higher visibility using phase contrast than fluorescence labeling, highlighting the capability of phase contrast to image these cell types (15).

#### 3.5.2 Microglia

**Figure 6:**
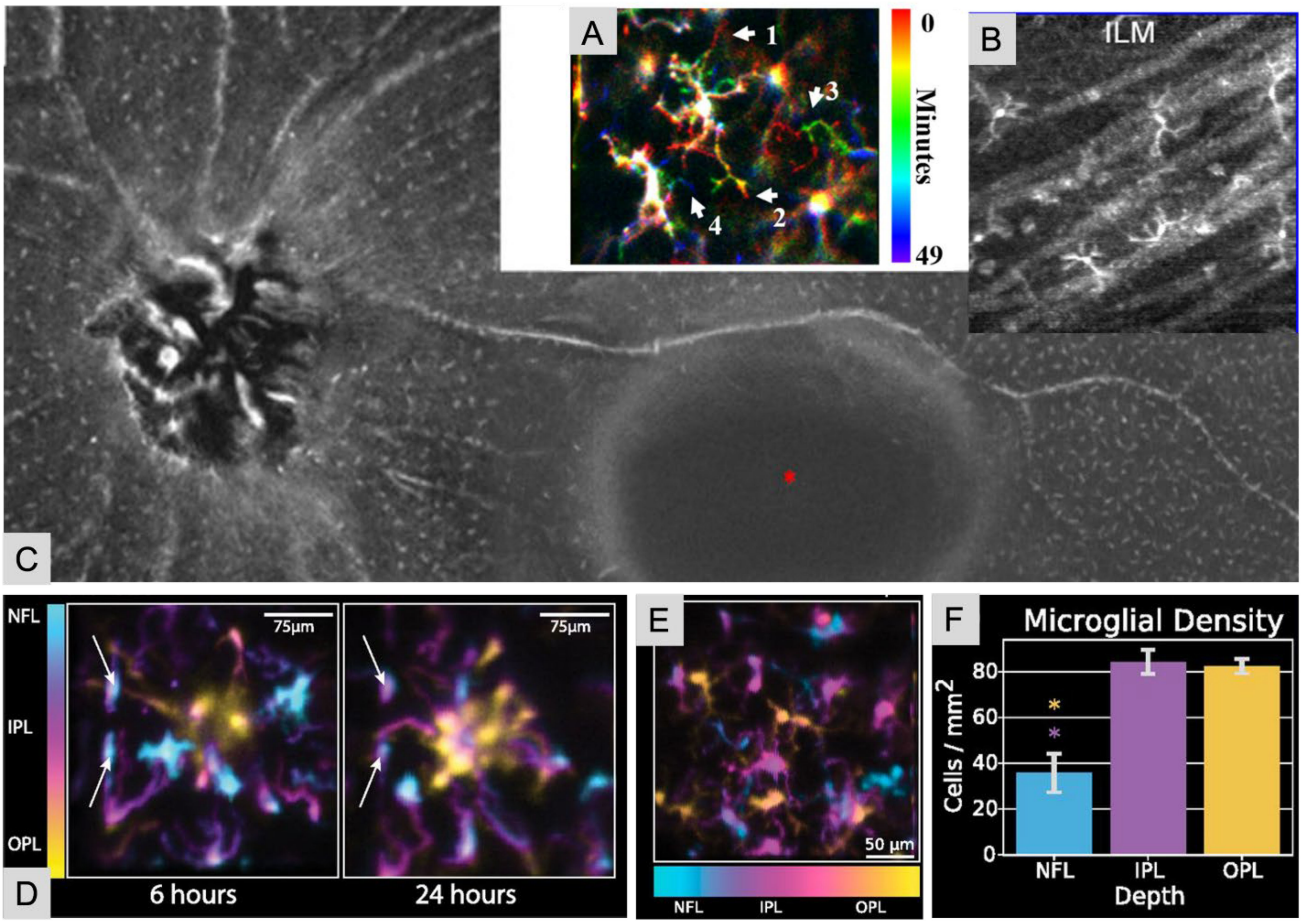
Images of microglial cells in the retina. **(A)***In vivo* AO-Confocal SLO fluorescence images of EGFP labeled microglia in mice. Microglia images were color-coded with time from 0 to 49 minutes shown in the scale bar. White arrows 1–4 note signify areas of growth and retraction. (Adapted with permission from Wahl et al., 2019). **(B)** Macrophage cells resolved above the ILM in cross-sectional views from averaged AO-OCT volumes in a healthy control subject. (Adapted with permission from Hammer et al., 2020). **(C)** Example of the spatial patterning of macrophage-like cells relative to the superficial retinal vasculature in a healthy subject. Shown is a montage of 3-μm *en face* OCT-R slabs located just above the ILM. (Reproduced from Castanos et al., 2020) **(D)** AO-SLO images revealing the three-dimensional migration of microglia at 6 and 24 hours after injury in a living mouse. **(E)** Depth-encoded AO-SLO image of microglia in a Cx3CR1+/GFP mouse, with color indicating axial position within the retinal layers. **(F)** Display of microglial cell density across the nerve fiber layer (NFL), inner plexiform layer (IPL), and outer plexiform layer (OPL), measured approximately 750 μm from the optic nerve head (ONH). Significant differences were observed between NFL and IPL (P = 7.45 × 10^−5) and NFL and OPL (P = 3.5 × 10^−5), with no difference between IPL and OPL (P = 0.77). *P < 0.0001. (Panels D-F reproduced with permission from Miller et al., 2019).

Studies of microglia in animal models often rely upon labeling techniques for visualization (16, 60); however, such techniques can be challenging as these cells share common markers with other macrophages and peripheral myeloid cells (16). In previous works, Wahl and colleagues imaged enhanced green fluorescent protein (EGFP)-labelled microglia in mice *in vivo* using a hill-climbing algorithm in a wavefront sensorless AO system (266). Later, Wahl and colleagues discussed a multimodal sensorless AO imaging system that combines OCT, OCTA, confocal SLO, and fluorescence detection, demonstrating high resolution, *in vivo* time-lapse and volumetric imaging of fluorescent-labelled microglia in mice (**Figure 6A**) (36). They also observed microglia branching with sensorless AO-SLO (36). Zawadzki et al. combined AO-SLO and phase-variance OCT/widefield SLO to localize microglia in 3D in the live mouse retina (267). AO-SLO images were registered to precise axial planes provided by phase variance OCT and widefield SLO, allowing for direct localization of microglia in specific retinal layers (267).

Label-free imaging techniques have also been explored. Gofas-Salas et al. recently described a radial multi-offset detection pattern combined with AO-SLO to image human retinal microglia (223). Hammer et al. utilized label-free AO-OCT to describe microglial spatial distribution in human eyes *in vivo* (60). Notably, these authors focused on the cells visible above the ILM, which may be more easily targeted without labelling techniques (**Figure 6B**) (60). Interestingly, Hammer et al. suggested that human microglia may have different dynamic characteristics compared to those seen in animal models (60). Human macrophages were also more likely to be seen in the periphery than in the central macula in healthy eyes (**Figure 6C**) (60, 268).

Further, much effort has focused on the imaging of microglial cellular dynamics. As a part of their immune function, microglial cells have highly dynamic processes which aid in continuous environmental surveillance (16). In a recent review, Eme-Scolan and Dando describe the imaging tools that can be used to observe live, dynamic behaviors of retinal microglial cells *in vivo (*16*).* AO-SLO has been used to describe the spatial distribution, dynamic behavior, and morphology of retinal microglia (16, 17). Confocal SLO has been used to quantify microglial dynamics following laser injury in mice retinas *in vivo* (269–272). Miller et al. recently used an AO-SLO and SLO-OCT system to demonstrate microglial volumetric distribution and dynamic behavior in the days and weeks following laser injury in mice retinas *in vivo* (**Figures 6D-F**) (17). In addition, Qin et al. developed an AO-two photon excitation fluorescence microscopy system which used a non-linear fluorescent guide star to visualize time-lapse dynamics of microglial behavior at subcellular resolution in the live mouse retina (197). They demonstrated that this imaging method can be used to characterized microglia dynamics and suggested that further application of this methodology may provide insight into cell interactions within the retinal microenvironment (197). Mezu-Ndubuisi et al. utilized fluorescein angiography, SD-OCT, and focal electroretinography to assess gliosis and microglia activation in mice models of oxygen-induced ischemic retinopathy (273). They noted retinal thinning and inner retinal dysfunction on imaging, while histological assessment demonstrated gliosis, tissue disorganization, and ectopic rod-bipolar cell synapses (273).

Imaging retinal microglia dynamics in human eyes has also been investigated. Kurokawa et al. observed retinal dynamics in human eyes *in vivo* across a variety of time intervals including seconds, minutes, and one year (274). Importantly, these authors developed novel post-capture processing methods involving temporal correlation; they corrected for motion artifacts in subcellular-resolution images through volumetric B-scan registration and time averaging AO-OCT volumes (274). Kurokawa et al. also reported temporal dynamics of hyalocytes, a macrophage-like cell just anterior to the ILM in the cortical vitreous, providing evidence that AO-OCT can be used to track *in vivo* function motion of these cells as they sample the retinal environment (274). Separately, Rui et al. designed a fiber-bundle with a central confocal fiber surrounded by six fibers to obtain multi-offset AO-SLO imaging of one focal plane (57). With this device, human microglial movement was quantified in both healthy and inflammatory disease states. Compared to previous iterations of this technology, the fiber bundle-AO-SLO allowed for dynamic visualization over short time intervals (57).

Some authors have suggested that glial cell mediated neurotoxicity plays a role in the pathogenesis of glaucoma, making imaging of these cells and their functional characteristics over time of particular interest to this disease process (60, 275). In retinal vascular diseases, gliosis occurs prior to vascular leakage and damage can be seen on fluorescent angiography (276). Glial activation can precede the onset of retinopathy and is thought to affect a variety of cell types including horizontal, amacrine, ganglion and photoreceptor cells (275, 276). Kumar and Zhuo utilized a transgenic mouse model of diabetic retinopathy to observe gliosis using confocal SLO in a live genetically engineered mouse model (276). These authors noted progressive astrocyte hyperplasia that preceded vascular structural changes (276). Similarly, Bosco et al. utilized live imaging confocal SLO to image the dynamics of fluorescent microglia in a murine model of glaucoma (275). Mice that exhibited increased and early microglial activation tended to have a higher severity of optic nerve damage (275). Overall, these authors noted that confocal SLO was able to track gliosis over time and establish this cell type as an indicator and predictor of prognostic outcomes (275). Interestingly, they documented variable spatial patterns of microglial activation and suggested that these cells may be responding to focal retinal changes during the earliest disease stages (275). In the future, high resolution, *in vivo* imaging may be useful clinically in detecting early neurodegenerative progression (275), which may inform treatment planning or alterations in treatment course.

#### 3.5.3 Müller cells

**Figure 7:**
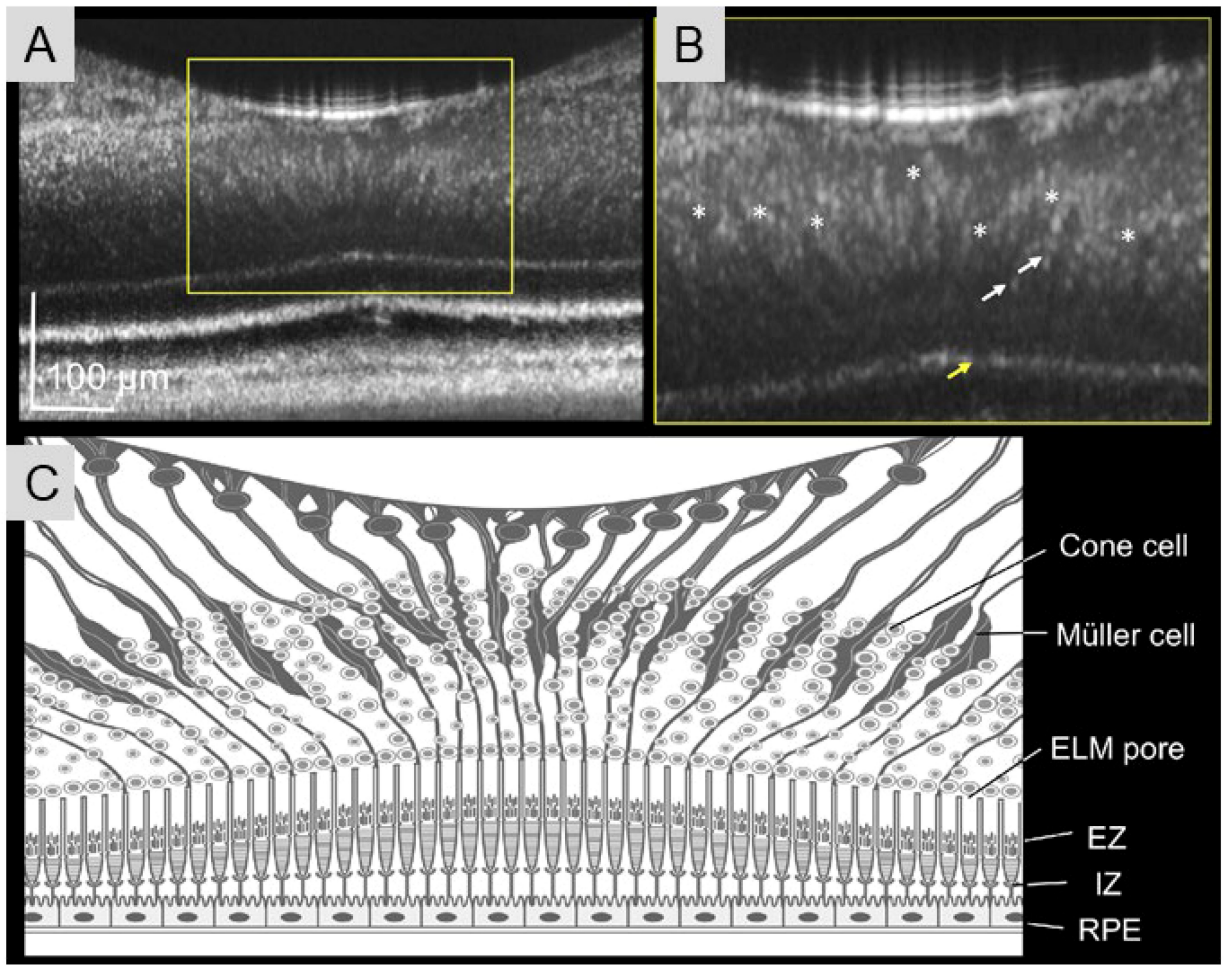
AO-OCT images of Müller cells in the human fovea. The AO-OCT image **(A)** and a magnified subregion (yellow square, **(B)** depicting Müller cells spanning diagonally from the inner limiting membrane to external limiting membrane (ELM) (ELM = asterisk). **(C)** AO-OCT foveal image, illustrating distinctive structural features of Müller cells. Also depicted are highly reflective dots within the outer nuclear layer (white arrow in B, likely cone nuclei) and small pores in the ELM (yellow arrow in B, likely the openings between adjacent Müller cell–photoreceptor junctions). (Adapted and reproduced with permission from Kadomoto et al., 2021).

Müller cells are a type of glial cell located in the inner retina and function in a variety of regulations in the retina (277). Müller cell dysfunction has been linked to the neuronal excitotoxicity and neurodegeneration seen in retinal diseases (277). Previously, Prasse et al. imaged fluorescently stained Müller cells with excitation at 633nm in whole mount monkey and human retinas, demonstrating that these cells are not light-reflecting but may also be light-guiding (278). Similar results have been found in Guinea pig retinas (279). However, *in vivo* studies of Müller cells are limited. Recently, AO-OCT has also been used to view foveal Müller cells in healthy human eyes with resolution of 3.4-µm and 3-µm in the axial and transverse dimensions, respectively (**Figure 7**) (280). Zhang et al. described an OCT-confocal SLO system capable of simultaneous cellular resolution images of *in vivo* Müller cells and microglia, even with expression of different fluorophores (247). In a recent pilot study, Arrigo et al. utilized structural OCT data to detect and quantify peripheral Müller cells in the human retina *in vivo* (281). Their results were similar to the histological findings in imaged patients requiring enucleation. These authors note that this technique is limited to a resolution of 8-µm, which may be more useful in identifying peripheral Müller cells compared to those in the foveal region (281).

## 4 Discussion

In summary, *in vivo* imaging techniques have significantly progressed over the last several decades, affording the ability to view cellular and sub-cellular detail in living models. Such improved capabilities have greatly facilitated investigations in ophthalmology and vision research. For instance, Azimipour et al. measured light-evoked, functional responses of human rods and cones (215). Others have proposed that imaging the processes of the visual cycle may aid in the quantification of rod and cone function (19). Retinoids can be visualized with two-photon excitation, providing insight into the visual cycle and photoreceptor function (264). Further, healthy eyes have been shown to transiently increase the length of their outer segments in response to light exposure; however, this process is thought to decrease retinal diseases. Lassoued and colleagues combined AO and phase-sensitive OCT to measure changes in optical path length inside cones in models of retinitis pigmentosa (222). This technique obtained 3D reflectance profiles at single cone resolution and was able to changes in optical path length with a sensitivity of 5nm (222). The cellular resolution of this technique is of particular importance for diseases that cause degeneration in individual photoreceptor cells, as changes at the cellular level can be noted prior to gross changes in cell density (222).

In addition, *in vivo* cellular resolution has already shown positive impact for early diagnosis of retinal diseases. Bosco et al. imaged a mouse model of inherited glaucoma using confocal SLO *in vivo* (275). These authors determined that microgliosis at the optic nerve head may be an indicator for ganglion cell stress and damage (275). The investigation of microglia behavior in various disease states provides the opportunity to investigate their role in disease potentiation, as well as potential therapeutic markers (60, 276). The ability to effectively image the microenvironment of these cells provides the opportunity to monitor therapy at the cellular level and discover which drugs may successfully intervene at earlier timepoints in the pathogenic process. Recent improvements in AO have allowed fundus cameras, SLO and OCT to capture near cellular resolution images of photoreceptors, ganglion cells and microvasculature. Clinically, this facilitates diagnosis, management and monitoring of various ocular conditions like AMD, diabetic retinopathy and glaucoma, allowing physicians to detect microscopic structural changes and with more precise monitoring of disease progression and therapeutic response (282–284). For example, advancements in AO-2PFM have allowed researchers to observe the effect of lidocaine administration on suppressing RGC hyperactivity, demonstrating its potential in evaluating pharmacological therapies and tracking progression of retinal diseases (68). In combination with fluorescent SLO imaging, as a surrogate for amyloid-beta (Aβ) plaque quantification in the brain, Sidiqi et al. found that retinal Aβ levels correlated with those in the cortex and were elevated in older mice (285). Interestingly, Aβ was more likely to be found in inner retinal layers with some found inside RGCs (285).

Although *in vivo* cellular-resolution imaging is advancing rapidly, the interpretative certainty of many techniques remains limited by incomplete histological correlation. For some modalities, such as AO-SLO cone mosaics, OCT lamination, and TPM-based structural imaging, extensive validation in human donor tissue or in animal models provides strong confidence in cellular identity. However, for several emerging high-resolution approaches, including vis-OCT, AO-OCT, and dynamic-contrast OCT, the biological interpretation of observed structures is still partly inferential and based on optical contrast mechanisms rather than direct one-to-one histological confirmation. This gap highlights an important direction for future work: integrating *in vivo* imaging with post-mortem tissue analysis or genetically targeted labeling to rigorously validate cellular and subcellular features. Addressing this limitation will be essential for translating these technologies into clinical biomarkers.

The future directions of *in vivo* cellular imaging of the retina are poised to revolutionize our understanding of ocular health and diseases. Advancements in this field are expected to focus on enhancing resolution, improving functional imaging, and integrating artificial intelligence (AI) for better diagnosis and management of retinal diseases. For instance, Lee et al. developed an advanced SS-OCT with computational aberration correction capable of attaining wide-field, 3D, cellular resolution imaging of photoreceptors, nerve fiber layers, and capillaries, which aids in the identification of early biomarkers in retinal disease (217). Additionally, phase-sensitive OCT images have shown capable of observing 10nm changes in tissue movement, providing insights into the functional responses of retinal cells, particularly photoreceptors (222, 231). As mentioned above, ORG is an emerging, non-invasive imaging modality capable of capturing retinal neuronal function (221, 231), and has recently been shown to differentiate cone classes and generate density maps with greater speed and accuracy compared to previous methods (208, 231). By combining different imaging modalities, such as OCT, fundus photography, and retinal fluorescein angiography, a more comprehensive view of the retina can be achieved. This approach can provide valuable information on both structural and functional aspects of retinal health. By analyzing vast datasets from retinal images, AI algorithms can assist in identifying subtle patterns that may indicate early stages of disease, which might be missed by the human eye. This could lead to automated, highly accurate diagnostic systems that can predict and prevent severe ocular conditions. For instance, AI analysis of fundus photos, OCT and OCT angiography is capable of screening and providing early diagnoses and management of various ocular conditions such as diabetic retinopathy and AMD (286–288). Telemedicine and remote imaging technologies will also become more prevalent, making retinal imaging more accessible, especially in underserved areas. Portable, user-friendly imaging devices, coupled with cloud-based AI diagnostics, could democratize eye care, allowing for frequent, non-invasive monitoring of retinal health. All of these advancements are heading towards more sophisticated, non-invasive, and comprehensive diagnostic methods to not only enhance our understanding of retinal diseases but also pave the way for more personalized and effective eye care.

## References

[1] London A Benhar I Schwartz M . The retina as a window to the brain—from eye research to CNS disorders. Nat Rev Neurol. (2013) 9:44–53. doi: 10.1038/nrneurol.2012.227, PMID: 23165340

[2] Rossi EA Granger CE Sharma R Yang Q Saito K Schwarz C . Imaging individual neurons in the retinal ganglion cell layer of the living eye. Proc Natl Acad Sci. (2017) 114:586–91. doi: 10.1073/pnas.1613445114, PMID: 28049835 PMC5255596

[3] Travis GH Golczak M Moise AR Palczewski K . Diseases caused by defects in the visual cycle: retinoids as potential therapeutic agents. Annu Rev Pharmacol Toxicol. (2007) 47:469–512. doi: 10.1146/annurev.pharmtox.47.120505.105225, PMID: 16968212 PMC2442882

[4] Euler T Haverkamp S Schubert T Baden T . Retinal bipolar cells: elementary building blocks of vision. Nat Rev Neurosci. (2014) 15:507–19. doi: 10.1038/nrn3783, PMID: 25158357

[5] Kiser PD Golczak M Maeda A Palczewski K . Key enzymes of the retinoid (visual) cycle in vertebrate retina. Biochim Biophys Acta (BBA) - Mol Cell Biol Lipids. (2012) 1821:137–51. doi: 10.1016/j.bbalip.2011.03.005, PMID: 21447403 PMC3158816

[6] Tsin A Betts-Obregon B Grigsby J . Visual cycle proteins: structure, function, and roles in human retinal disease. J Biol Chem. (2018) 293:13016–21. doi: 10.1074/jbc.AW118.003228, PMID: 30002120 PMC6109927

[7] Wang J-S Kefalov VJ . The Cone-specific visual cycle. Prog Retinal Eye Res. (2011) 30:115–28. doi: 10.1016/j.preteyeres.2010.11.001, PMID: 21111842 PMC3073571

[8] Rando RR . The biochemistry of the visual cycle. Chem Rev. (2001) 101:1881–96. doi: 10.1021/cr960141c, PMID: 11710234

[9] Li W DeVries SH . Bipolar cell pathways for color and luminance vision in a dichromatic mammalian retina. Nat Neurosci. (2006) 9:669–75. doi: 10.1038/nn1686, PMID: 16617341

[10] Kolb H . Amacrine cells of the mammalian retina: Neurocircuitry and functional roles. Eye. (1997) 11:904–23. doi: 10.1038/eye.1997.230, PMID: 9537156

[11] Mure LS . Intrinsically photosensitive retinal ganglion cells of the human retina. Front Neurol. (2021) 12. doi: 10.3389/fneur.2021.636330, PMID: 33841306 PMC8027232

[12] Kwon W Freeman SA . Phagocytosis by the retinal pigment epithelium: recognition, resolution, recycling. Front Immunol. (2020) 11. doi: 10.3389/fimmu.2020.604205, PMID: 33281830 PMC7691529

[13] Strauss O . The retinal pigment epithelium. In: Webvision: The Organization of the Retina and Visual System. Salt Lake City (UT): University of Utah Health Sciences Center (2011). 21413389

[14] Boya P Codogno P . Recycling in sight. Nature. (2013) 501:40–2. doi: 10.1038/501040a, PMID: 24005411

[15] Joseph A Chu CJ Feng G Dholakia K Schallek J . Label-free imaging of immune cell dynamics in the living retina using adaptive optics. Elife. (2020) 9:e60547. doi: 10.7554/eLife.60547.sa2 33052099 PMC7556865

[16] Eme-Scolan E Dando SJ . Tools and approaches for studying microglia *in vivo*. Front Immunol. (2020) 11:583647. doi: 10.3389/fimmu.2020.583647, PMID: 33117395 PMC7576994

[17] Miller EB Zhang P Ching K Pugh EN Jr. Burns ME . *In vivo* imaging reveals transient microglia recruitment and functional recovery of photoreceptor signaling after injury. Proc Natl Acad Sci. (2019) 116:16603–12. doi: 10.1073/pnas.1903336116, PMID: 31350349 PMC6697899

[18] O’Koren E Mathew R Saban D . Fate mapping reveals that microglia and recruited monocyte-derived macrophages are definitively distinguishable by phenotype in the retina. Sci Rep. (2016) 6:1–12. doi: 10.1038/srep20636, PMID: 26856416 PMC4746646

[19] Hunter JJ Merigan WH Schallek JB . Imaging retinal activity in the living eye. Annu Rev Vision Sci. (2019) 5:15. doi: 10.1146/annurev-vision-091517-034239, PMID: 31525142 PMC7001891

[20] Zhong Z Huang G Chui TYP Petrig BL Burns SA . Local flicker stimulation evokes local retinal blood velocity changes. J Vision. (2012) 12:3–3. doi: 10.1167/12.6.3, PMID: 22661609 PMC3410729

[21] Zhang L Liu W Wang H-Y Qiang W Wang R Cui Z-L . The temporal progression of retinal degeneration and early-stage idebenone treatment in the Pde6brd1/rd1 mouse model of retinal dystrophy. Sci Rep. (2024) 14:2019. doi: 10.1038/s41598-024-52391-y, PMID: 38263197 PMC10805728

[22] Mansour AM Gad MS Habib S Elmasry K . Bidirectional hypoxic extracellular vesicle signaling between müller glia and retinal pigment epithelium regulates retinal metabolism and barrier function. Biology. (2025) 14:1014. doi: 10.3390/biology14081014, PMID: 40906190 PMC12383891

[23] Wang S Li W Chen M Cao Y Lu W Li X . The retinal pigment epithelium: functions and roles in ocular diseases. Fundam Res. (2024) 4:1710–8. doi: 10.1016/j.fmre.2023.08.011, PMID: 39734536 PMC11670733

[24] Zhang Q Gong D Huang M Zhu Z Yang W Ma G . Recent advances and applications of optical coherence tomography angiography in diabetic retinopathy. Front Endocrinol. (2025) 16:1438739. doi: 10.3389/fendo.2025.1438739, PMID: 40309445 PMC12040626

[25] Li R Zhang J Wu J-M Fan J-Q Lin B . Temporal biphasic regulation of photoreceptor degeneration by microglial TREM2: A metabolic-immune nexus in retinitis pigmentosa. Sci Adv. (2025) 11:eadw9299. doi: 10.1126/sciadv.adw9299, PMID: 40938987 PMC13141908

[26] Navneet S Wilson K Rohrer B . Müller glial cells in the macula: their activation and cell-cell interactions in age-related macular degeneration. Invest Ophthalmol Visual Sci. (2024) 65:42–2. doi: 10.1167/iovs.65.2.42, PMID: 38416457 PMC10910558

[27] Zhao N Hao X-N Huang J-M Song Z-M Tao Y . Crosstalk between microglia and Müller glia in the age-related macular degeneration: role and therapeutic value of neuroinflammation. Aging Dis. (2024) 15:1132. doi: 10.14336/AD.2023.0823-3, PMID: 37728589 PMC11081163

[28] Jeon C-J Strettoi E Masland RH . The major cell populations of the mouse retina. J Neurosci. (1998) 18:8936. doi: 10.1523/JNEUROSCI.18-21-08936.1998, PMID: 9786999 PMC6793518

[29] Mateos JM Barmettler G Doehner J Naharros IO Guhl B Neuhauss SC . Correlative super-resolution and electron microscopy to resolve protein localization in zebrafish retina. JoVE (Journal Visualized Experiments). (2017):e56113. doi: 10.3791/56113, PMID: 29155784 PMC5755354

[30] Haverkamp S Wässle H . Immunocytochemical analysis of the mouse retina. J Comp Neurol. (2000) 424:1–23. doi: 10.1002/1096-9861(20000814)424:1<1::AID-CNE1>3.0.CO;2-V 10888735

[31] Cuenca N Ortuño-Lizarán I Pinilla I . Cellular characterization of OCT and outer retinal bands using specific immunohistochemistry markers and clinical implications. Ophthalmology. (2018) 125:407–22. doi: 10.1016/j.ophtha.2017.09.016, PMID: 29037595

[32] Dvoriantchikova G Ivanov D Panchin Y Shestopalov VI . Expression of pannexin family of proteins in the retina. FEBS Lett. (2006) 580:2178–82. doi: 10.1016/j.febslet.2006.03.026, PMID: 16616526

[33] Das Y Roose N De Groef L Fransen M Moons L Van Veldhoven PP . Differential distribution of peroxisomal proteins points to specific roles of peroxisomes in the murine retina. Mol Cell Biochem. (2019) 456:53–62. doi: 10.1007/s11010-018-3489-3, PMID: 30604065

[34] Hollenberg MJ Lea PJ . High resolution scanning electron microscopy of the retinal pigment epithelium and Bruch’s layer. Invest Ophthalmol Visual Sci. (1988) 29:1380–9. 3417422

[35] Huber G Beck SC Grimm C Sahaboglu-Tekgoz A Paquet-Durand F Wenzel A . Spectral domain optical coherence tomography in mouse models of retinal degeneration. Invest Ophthalmol Visual Sci. (2009) 50:5888–95. doi: 10.1167/iovs.09-3724, PMID: 19661229 PMC2800101

[36] Wahl DJ Ng R Ju MJ Jian Y Sarunic MV . Sensorless adaptive optics multimodal en-face small animal retinal imaging. Biomed Optics Express. (2019) 10:252–67. doi: 10.1364/BOE.10.000252, PMID: 30775098 PMC6363194

[37] Keeler CR . The ophthalmoscope in the lifetime of Hermann von Helmholtz. Arch Ophthalmol. (2002) 120:194–201. doi: 10.1001/archopht.120.2.194, PMID: 11831923

[38] Schett A . The discovery of the ophthalmoscope by Hermann von Helmholtz (1821-1894). Strabismus. (1999) 7:241–4. doi: 10.1076/stra.7.4.241.626, PMID: 10819608

[39] Patton N Aslam TM MacGillivray T Deary IJ Dhillon B Eikelboom RH . Retinal image analysis: Concepts, applications and potential. Prog Retinal Eye Res. (2006) 25:99–127. doi: 10.1016/j.preteyeres.2005.07.001, PMID: 16154379

[40] Nakagawa T Suzuki T Hayashi Y Mizukusa Y Hatanaka Y Ishida K . Quantitative depth analysis of optic nerve head using stereo retinal fundus image pair. J Biomed Optics. (2008) 13:064026. doi: 10.1117/1.3041711, PMID: 19123672

[41] Jain N Farsiu S Khanifar AA Bearelly S Smith RT Izatt JA . Quantitative comparison of drusen segmented on SD-OCT versus drusen delineated on color fundus photographs. Invest Ophthalmol Visual Sci. (2010) 51:4875–83. doi: 10.1167/iovs.09-4962, PMID: 20393117 PMC2939301

[42] Sharp PF Manivannan A Xu H Forrester JV . The scanning laser ophthalmoscope—a review of its role in bioscience and medicine. Phys Med Biol. (2004) 49:1085. doi: 10.1088/0031-9155/49/7/001, PMID: 15128191

[43] Spaide RF Klancnik JM Jr Cooney MJ . Retinal vascular layers imaged by fluorescein angiography and optical coherence tomography angiography. JAMA Ophthalmol. (2015) 133:45–50. doi: 10.1001/jamaophthalmol.2014.3616, PMID: 25317632

[44] Patel M Kiss S . Ultra-wide-field fluorescein angiography in retinal disease. Curr Opin Ophthalmol. (2014) 25:213–20. doi: 10.1097/ICU.0000000000000042, PMID: 24614144

[45] Mendis KR Balaratnasingam C Yu P Barry CJ McAllister IL Cringle SJ . Correlation of histologic and clinical images to determine the diagnostic value of fluorescein angiography for studying retinal capillary detail. Invest Ophthalmol Visual Sci. (2010) 51:5864–9. doi: 10.1167/iovs.10-5333, PMID: 20505200

[46] Manivannan A Plskova J Farrow A McKay S Sharp PF Forrester JV . Ultra-wide-field fluorescein angiography of the ocular fundus. Am J Ophthalmol. (2005) 140:525–7. doi: 10.1016/j.ajo.2005.02.055, PMID: 16139004

[47] Blair MP Shapiro MJ Hartnett ME . Fluorescein angiography to estimate normal peripheral retinal nonperfusion in children. J Am Assoc Pediatr Ophthalmol Strabismus. (2012) 16:234–7. doi: 10.1016/j.jaapos.2011.12.157, PMID: 22681939 PMC3756139

[48] Yannuzzi LA . Indocyanine green angiography: a perspective on use in the clinical setting. Am J Ophthalmol. (2011) 151:745–751. e741. doi: 10.1016/j.ajo.2011.01.043, PMID: 21501704

[49] Huang D Swanson EA Lin CP Schuman JS Stinson WG Chang W . Optical coherence tomography. science. (1991) 254:1178–81. doi: 10.1126/science.1957169, PMID: 1957169 PMC4638169

[50] Drexler W Fujimoto JG . State-of-the-art retinal optical coherence tomography. Prog Retinal Eye Res. (2008) 27:45–88. doi: 10.1016/j.preteyeres.2007.07.005, PMID: 18036865

[51] van Velthoven MEJ Faber DJ Verbraak FD van Leeuwen TG de Smet MD . Recent developments in optical coherence tomography for imaging the retina. Prog Retinal Eye Res. (2007) 26:57–77. doi: 10.1016/j.preteyeres.2006.10.002, PMID: 17158086

[52] Sakata LM DeLeon-Ortega J Sakata V Girkin CA . Optical coherence tomography of the retina and optic nerve – a review. Clin Exp Ophthalmol. (2009) 37:90–9. doi: 10.1111/j.1442-9071.2009.02015.x, PMID: 19338607

[53] Costa RA Skaf M Melo LAS Calucci D Cardillo JA Castro JC . Retinal assessment using optical coherence tomography. Prog Retinal Eye Res. (2006) 25:325–53. doi: 10.1016/j.preteyeres.2006.03.001, PMID: 16716639

[54] Röhlig M Schmidt C Prakasam RK Rosenthal P Schumann H Stachs O . Visual analysis of retinal changes with optical coherence tomography. Visual Comput. (2018) 34:1209–24. doi: 10.1007/s00371-018-1486-x

[55] Hariri S Moayed AA Dracopoulos A Hyun C Boyd S Bizheva K . Limiting factors to the OCT axial resolution for *in-vivo* imaging of human and rodent retina in the 1060nm wavelength range. Opt Express. (2009) 17:24304–16. doi: 10.1364/OE.17.024304, PMID: 20052141

[56] Liba O Lew MD SoRelle ED Dutta R Sen D Moshfeghi DM . Speckle-modulating optical coherence tomography in living mice and humans. Nat Commun. (2017) 8:15845. doi: 10.1038/ncomms15845, PMID: 28632205 PMC5481831

[57] Rui Y Lee DMW Zhang M Snyder VC Raghuraman R Gofas-Salas E . Imaging the structure and dynamic activity of retinal microglia and macrophage-like cells in the living human eye. bioRxiv. (2022) 2022:2004.2030.490173. doi: 10.1101/2022.04.30.490173

[58] Akyol E Hagag AM Sivaprasad S Lotery AJ . Adaptive optics: principles and applications in ophthalmology. Eye. (2021) 35:244–64. doi: 10.1038/s41433-020-01286-z, PMID: 33257798 PMC7852593

[59] Burns SA Elsner AE Sapoznik KA Warner RL Gast TJ . Adaptive optics imaging of the human retina. Prog Retinal Eye Res. (2019) 68:1–30. doi: 10.1016/j.preteyeres.2018.08.002, PMID: 30165239 PMC6347528

[60] Hammer DX Agrawal A Villanueva R Saeedi O Liu Z . Label-free adaptive optics imaging of human retinal macrophage distribution and dynamics. Proc Natl Acad Sci. (2020) 117:30661–9. doi: 10.1073/pnas.2010943117, PMID: 33168747 PMC7720180

[61] Jian Y Xu J Gradowski MA Bonora S Zawadzki RJ Sarunic MV . Wavefront sensorless adaptive optics optical coherence tomography for *in vivo* retinal imaging in mice. Biomed Optics Express. (2014) 5:547–59. doi: 10.1364/BOE.5.000547, PMID: 24575347 PMC3920883

[62] Ju MJ Huang C Wahl DJ Jian Y Sarunic MV . Visible light sensorless adaptive optics for retinal structure and fluorescence imaging. Optics Lett. (2018) 43:5162–5. doi: 10.1364/OL.43.005162, PMID: 30320845

[63] Wynne N Carroll J Duncan JL . Promises and pitfalls of evaluating photoreceptor-based retinal disease with adaptive optics scanning light ophthalmoscopy (AOSLO). Prog Retinal Eye Res. (2021) 83:100920. doi: 10.1016/j.preteyeres.2020.100920, PMID: 33161127 PMC8639282

[64] Liu Z Aghayee S Soltanian-Zadeh S Kovalick K Agrawal A Saeedi O . Quantification of human photoreceptor–retinal pigment epithelium macular topography with adaptive optics–optical coherence tomography. Diagnostics. (2024) 14:1518. doi: 10.3390/diagnostics14141518, PMID: 39061655 PMC11276449

[65] Luo S Wong JH Roorda A . *In-vivo* imaging of human retinal ganglion cells using real-time tracked adaptive optics optical coherence tomography. Invest Ophthalmol Visual Sci. (2025) 66:999–9.

[66] Zhang F Kovalick K Raghavendra A Soltanian-Zadeh S Farsiu S Hammer DX . *In vivo* imaging of human retinal ganglion cells using optical coherence tomography without adaptive optics. Biomed Optics Express. (2024) 15:4675–88. doi: 10.1364/BOE.533249, PMID: 39346995 PMC11427184

[67] Lee DM Zhang M Snyder VC Rossi EA . Multi-spectral autofluorescence variability of the individual retinal pigmented epithelial cells in healthy aging eyes. Sci Rep. (2024) 14:30012. doi: 10.1038/s41598-024-81433-8, PMID: 39622926 PMC11612473

[68] Zhang Q Yang Y Cao KJ Chen W Paidi S Xia C-h . Retinal microvascular and neuronal pathologies probed *in vivo* by adaptive optical two-photon fluorescence microscopy. Elife. (2023) 12:e84853. doi: 10.7554/eLife.84853.sa2, PMID: 37039777 PMC10089658

[69] Hong J Chen S Pohodich A Gill T Song X Jian Y . Convolutional neural network-based differentiation of intraocular inflammatory cells with ultrahigh-resolution OCT. Biomed Optics Express. (2025) 16:4917–28. doi: 10.1364/BOE.575636, PMID: 41394506 PMC12698106

[70] Han L Mupparapu R Chrysostomou V Bell KC Chong SP Chen J . Longitudinal structural and microvascular imaging of mouse retina after optic nerve crush using temporal speckle-averaging visible light OCT. Invest Ophthalmol Visual Sci. (2025) 66:56–6. doi: 10.1167/iovs.66.13.56, PMID: 41171041 PMC12582194

[71] Pi S Hormel TT Wei X Cepurna W Morrison JC Jia Y . Imaging retinal structures at cellular-level resolution by visible-light optical coherence tomography. Optics Lett. (2020) 45:2107–10. doi: 10.1364/OL.386454, PMID: 32236080 PMC8575555

[72] Britten-Jones AC Thai L Flanagan JP Bedggood PA Edwards TL Metha AB . Adaptive optics imaging in inherited retinal diseases: A scoping review of the clinical literature. Survey Ophthalmol. (2024) 69:51–66. doi: 10.1016/j.survophthal.2023.09.006, PMID: 37778667

[73] Song S Hormel TT Jia Y . Visible-light optical coherence tomography and its applications. Neurophotonics. (2025) 12:020601–1. doi: 10.1117/1.NPh.12.2.020601, PMID: 40206421 PMC11981582

[74] Mecê P Groux K Scholler J Thouvenin O Fink M Grieve K . Coherence gate shaping for wide field high-resolution *in vivo* retinal imaging with full-field OCT. Biomed Optics Express. (2020) 11:4928–41. doi: 10.1364/BOE.400522, PMID: 33014591 PMC7510855

[75] Merkle CW Leahy C Srinivasan VJ . Dynamic contrast optical coherence tomography images transit time and quantifies microvascular plasma volume and flow in the retina and choriocapillaris. Biomed Optics Express. (2016) 7:4289–312. doi: 10.1364/BOE.7.004289, PMID: 27867732 PMC5102529

[76] Monfort T Azzollini S Brogard J Clémençon M Slembrouck-Brec A Forster V . Dynamic full-field optical coherence tomography module adapted to commercial microscopes allows longitudinal *in vitro* cell culture study. Commun Biol. (2023) 6:992. doi: 10.1038/s42003-023-05378-w, PMID: 37770552 PMC10539404

[77] Palczewska G Wojtkowski M Palczewski K . From mouse to human: Accessing the biochemistry of vision *in vivo* by two-photon excitation. Prog Retinal Eye Res. (2023) 93:101170. doi: 10.1016/j.preteyeres.2023.101170, PMID: 36787681 PMC10463242

[78] Yannuzzi LA . The retinal atlas. Philadelphia, PA: Elsevier Health Sciences (2010).

[79] Hansell P Beeson EJG . Retinal photography in colour. Br J Ophthalmol. (1953) 37:65. doi: 10.1136/bjo.37.2.65, PMID: 13032356 PMC1324062

[80] Tran K Mendel TA Holbrook KL Yates PA . Construction of an inexpensive, hand-held fundus camera through modification of a consumer “point-and-shoot” camera. Invest Ophthalmol Visual Sci. (2012) 53:7600–7. doi: 10.1167/iovs.12-10449, PMID: 23049089 PMC3495602

[81] Yogesan K Constable IJ Barry CJ Eikelboom RH Morgan W Tay-Kearney M-L . Evaluation of a portable fundus camera for use in the teleophthalmologic diagnosis of glaucoma. J Glaucoma. (1999) 8:297–301. doi: 10.1097/00061198-199910000-00004, PMID: 10529928

[82] De Bats F Nitenberg CV Fantino B Denis P Kodjikian L . Age-related macular degeneration screening using a nonmydriatic digital color fundus camera and telemedicine. Ophthalmologica. (2014) 231:172–6. doi: 10.1159/000356695, PMID: 24356326

[83] Klein R Klein BE Neider MW Hubbard LD Meuer SM Brothers RJ . Diabetic retinopathy as detected using ophthalmoscopy, a nonmyciriatic camera and a standard fundus camera. Ophthalmology. (1985) 92:485–91. doi: 10.1016/S0161-6420(85)34003-4, PMID: 4000642

[84] Webb RH Hughes GW Pomerantzeff O . Flying spot TV ophthalmoscope. Appl Optics. (1980) 19:2991–7. doi: 10.1364/AO.19.002991, PMID: 20234539

[85] Woon W Fitzke F Bird A Marshall J . Confocal imaging of the fundus using a scanning laser ophthalmoscope. Br J Ophthalmol. (1992) 76:470–4. doi: 10.1136/bjo.76.8.470, PMID: 1390528 PMC504319

[86] Webb RH Hughes GW Delori FC . Confocal scanning laser ophthalmoscope. Appl Optics. (1987) 26:1492–9. doi: 10.1364/AO.26.001492, PMID: 20454349

[87] Wollstein G Garway-Heath DF Hitchings RA . Identification of early glaucoma cases with the scanning laser ophthalmoscope. Ophthalmology. (1998) 105:1557–63. doi: 10.1016/S0161-6420(98)98047-2, PMID: 9709774

[88] Bartsch D-U Weinreb RN Zinser G Freeman WR . Confocal scanning infrared laser ophthalmoscopy for indocyanine green angiography. Am J Ophthalmol. (1995) 120:642–51. doi: 10.1016/S0002-9394(14)72211-1, PMID: 7485366

[89] Leung CK-s Lindsey JD Crowston JG Lijia C Chiang S Weinreb RN . Longitudinal profile of retinal ganglion cell damage after optic nerve crush with blue-light confocal scanning laser ophthalmoscopy. Invest Ophthalmol Visual Sci. (2008) 49:4898–902. doi: 10.1167/iovs.07-1447, PMID: 18441315 PMC5557086

[90] Stevenson SB Roorda A . @ in ophthalmic technologies XV Vol. 5688. Bellingham, WA: SPIE (2005) p. 145–51.

[91] Bedggood P Metha A . De-warping of images and improved eye tracking for the scanning laser ophthalmoscope. PloS One. (2017) 12:e0174617. doi: 10.1371/journal.pone.0174617, PMID: 28369065 PMC5378343

[92] Hammer DX Ferguson RD Magill JC White MA Elsner AE Webb RH . Compact scanning laser ophthalmoscope with high-speed retinal tracker. Appl Optics. (2003) 42:4621–32. doi: 10.1364/AO.42.004621, PMID: 12916631

[93] Sheehy CK Yang Q Arathorn DW Tiruveedhula P de Boer JF Roorda A . High-speed, image-based eye tracking with a scanning laser ophthalmoscope. Biomed Optics Express. (2012) 3:2611–22. doi: 10.1364/BOE.3.002611, PMID: 23082300 PMC3469984

[94] Manivannan A Van der Hoek J Vieira P Farrow A Olson J Sharp PF . Clinical investigation of a true color scanning laser ophthalmoscope. Arch Ophthalmol. (2001) 119:819–24. doi: 10.1001/archopht.119.6.819, PMID: 11405832

[95] Tearney GJ Jang I-K Kang D-H Aretz HT Houser SL Brady TJ . Porcine coronary imaging *in vivo* by optical coherence tomography. Acta Cardiol. (2000) 55:233–7. doi: 10.2143/AC.55.4.2005745, PMID: 11041121

[96] Schmitt J Yadlowsky M Bonner R . Subsurface imaging of living skin with optical coherence microscopy. Dermatology. (1995) 191:93–8. doi: 10.1159/000246523, PMID: 8520074

[97] Mogensen M Thrane L Jørgensen TM Andersen PE Jemec GB . OCT imaging of skin cancer and other dermatological diseases. J Biophoton. (2009) 2:442–51. doi: 10.1002/jbio.200910020, PMID: 19557752

[98] Evans JA Poneros JM Bouma BE Bressner J Halpern EF Shishkov M . Optical coherence tomography to identify intramucosal carcinoma and high-grade dysplasia in Barrett’s esophagus. Clin Gastroenterol Hepatol. (2006) 4:38–43. doi: 10.1016/S1542-3565(05)00746-9, PMID: 16431303 PMC2703582

[99] Hee MR Izatt JA Swanson EA Huang D Schuman JS Lin CP . Optical coherence tomography of the human retina. Arch Ophthalmol. (1995) 113:325–32. doi: 10.1001/archopht.1995.01100030081025, PMID: 7887846

[100] Jiao S Knighton R Huang X Gregori G Puliafito CA . Simultaneous acquisition of sectional and fundus ophthalmic images with spectral-domain optical coherence tomography. Optics Express. (2005) 13:444–52. doi: 10.1364/OPEX.13.000444, PMID: 19488371

[101] Leitgeb R Hitzenberger C Fercher AF . Performance of fourier domain vs. time domain optical coherence tomography. Optics Express. (2003) 11:889–94. doi: 10.1364/OE.11.000889, PMID: 19461802

[102] Wojtkowski M Leitgeb R Kowalczyk A Bajraszewski T Fercher AF . *In vivo* human retinal imaging by Fourier domain optical coherence tomography. J Biomed Optics. (2002) 7:457–63. doi: 10.1117/1.1482379, PMID: 12175297

[103] Choma MA Sarunic MV Yang C Izatt JA . Sensitivity advantage of swept source and Fourier domain optical coherence tomography. Optics Express. (2003) 11:2183–9. doi: 10.1364/OE.11.002183, PMID: 19466106

[104] Wojtkowski M Srinivasan V Fujimoto JG Ko T Schuman JS Kowalczyk A . Three-dimensional retinal imaging with high-speed ultrahigh-resolution optical coherence tomography. Ophthalmology. (2005) 112:1734–46. doi: 10.1016/j.ophtha.2005.05.023, PMID: 16140383 PMC1939719

[105] Wojtkowski M Srinivasan VJ Ko TH Fujimoto JG Kowalczyk A Duker JS . Ultrahigh-resolution, high-speed, Fourier domain optical coherence tomography and methods for dispersion compensation. Optics Express. (2004) 12:2404–22. doi: 10.1364/OPEX.12.002404, PMID: 19475077

[106] Yun S Tearney G Bouma B Park B de Boer JF . High-speed spectral-domain optical coherence tomography at 1.3 µm wavelength. Optics Express. (2003) 11:3598–604. doi: 10.1364/OE.11.003598, PMID: 19471496 PMC2713046

[107] Klein T Wieser W Reznicek L Neubauer A Kampik A Huber R . Multi-mhz retinal oct. Biomed Optics Express. (2013) 4:1890–908. doi: 10.1364/BOE.4.001890, PMID: 24156052 PMC3799654

[108] Schmitt J Lee S Yung K . An optical coherence microscope with enhanced resolving power in thick tissue. Optics Commun. (1997) 142:203–7. doi: 10.1016/S0030-4018(97)00280-0

[109] Boppart SA Bouma BE Pitris C Southern JF Brezinski ME Fujimoto JG . *In vivo* cellular optical coherence tomography imaging. Nat Med. (1998) 4:861–5. doi: 10.1038/nm0798-861, PMID: 9662382

[110] Fercher AF . Optical coherence tomography. J Biomed Optics. (1996) 1:157–73. doi: 10.1117/12.231361, PMID: 23014682

[111] Drexler W Morgner U Ghanta RK Kärtner FX Schuman JS Fujimoto JG . Ultrahigh-resolution ophthalmic optical coherence tomography. Nat Med. (2001) 7:502–7. doi: 10.1038/86589, PMID: 11283681 PMC1950821

[112] Drexler W Morgner U Kärtner F Pitris C Boppart S Li X . *In vivo* ultrahigh-resolution optical coherence tomography. Opt Lett. (1999) 24:1221–3. doi: 10.1364/OL.24.001221, PMID: 18073990

[113] Leitgeb R Drexler W Unterhuber A Hermann B Bajraszewski T Le T . Ultrahigh resolution Fourier domain optical coherence tomography. Opt Express. (2004) 12:2156–65. doi: 10.1364/OPEX.12.002156, PMID: 19475051

[114] Werkmeister RM Sapeta S Schmidl D Garhöfer G Schmidinger G Dos Santos VA . Ultrahigh-resolution OCT imaging of the human cornea. Biomed Optics Express. (2017) 8:1221–39. doi: 10.1364/BOE.8.001221, PMID: 28271013 PMC5330598

[115] Ge X Chen S Chen S Liu L . High resolution optical coherence tomography. J Lightwave Technol. (2021) 39:3824–35. doi: 10.1109/JLT.2021.3061606

[116] Kumar A Drexler W Leitgeb RA . Numerical focusing methods for full field OCT: a comparison based on a common signal model. Opt Express. (2014) 22:16061–78. doi: 10.1364/OE.22.016061, PMID: 24977860

[117] Shemonski ND South FA Liu Y-Z Adie SG Scott Carney P Boppart SA . Computational high-resolution optical imaging of the living human retina. Nat Photonics. (2015) 9:440–3. doi: 10.1038/nphoton.2015.102, PMID: 26877761 PMC4750047

[118] Zhang J Mazlin V Fei K Boccara AC Yuan J Xiao P . Time-domain full-field optical coherence tomography (TD-FF-OCT) in ophthalmic imaging. Ther Adv Chronic Dis. (2023) 14:20406223231170146. doi: 10.1177/20406223231170146, PMID: 37152350 PMC10161339

[119] Beaurepaire E Boccara AC Lebec M Blanchot L Saint-Jalmes H . Full-field optical coherence microscopy. Optics Lett. (1998) 23:244–6. doi: 10.1364/OL.23.000244, PMID: 18084473

[120] Dubois A Grieve K Moneron G Lecaque R Vabre L Boccara C . Ultrahigh-resolution full-field optical coherence tomography. Appl Optics. (2004) 43:2874–83. doi: 10.1364/AO.43.002874, PMID: 15143811

[121] Akiba M Maeda N Yumikake K Soma T Nishida K Tano Y . Ultrahigh-resolution imaging of human donor cornea using full-field optical coherence tomography. J Biomed Optics. (2007) 12:041202–041202-041207. doi: 10.1117/1.2764461, PMID: 17867791

[122] Ghouali W Grieve K Bellefqih S Sandali O Harms F Laroche L . Full-field optical coherence tomography of human donor and pathological corneas. Curr Eye Res. (2015) 40:526–34. doi: 10.3109/02713683.2014.935444, PMID: 25251769

[123] Xiao P Mazlin V Grieve K Sahel J-A Fink M Boccara AC . *In vivo* high-resolution human retinal imaging with wavefront-correctionless full-field OCT. Optica. (2018) 5:409–12. doi: 10.1364/OPTICA.5.000409

[124] Mazlin V Xiao P Scholler J Irsch K Grieve K Fink M . Real-time non-contact cellular imaging and angiography of human cornea and limbus with common-path full-field/SD OCT. Nat Commun. (2020) 11:1868. doi: 10.1038/s41467-020-15792-x, PMID: 32313067 PMC7171111

[125] Mecê P Scholler J Groux K Boccara C . High-resolution *in-vivo* human retinal imaging using full-field OCT with optical stabilization of axial motion. Biomed Optics Express. (2020) 11:492–504. doi: 10.1364/BOE.381398, PMID: 32010530 PMC6968740

[126] Scholler J Groux K Grieve K Boccara C Mecê P . Adaptive-glasses time-domain FFOCT for wide-field high-resolution retinal imaging with increased SNR. Optics Lett. (2020) 45:5901–4. doi: 10.1364/OL.403135, PMID: 33137028

[127] Povazay B Apolonski AA Unterhuber A Hermann B Bizheva KK Sattmann H . in coherence domain Optical Methods in Biomedical Science and Clinical Applications VI Vol. 4619. Bellingham, WA: SPIE (2002) p. 90–4.

[128] Lichtenegger A Harper DJ Augustin M Eugui P Muck M Gesperger J . Spectroscopic imaging with spectral domain visible light optical coherence microscopy in Alzheimer’s disease brain samples. Biomed Optics Express. (2017) 8:4007–25. doi: 10.1364/BOE.8.004007, PMID: 28966843 PMC5611919

[129] Yi J Chen S Shu X Fawzi AA Zhang HF . Human retinal imaging using visible-light optical coherence tomography guided by scanning laser ophthalmoscopy. Biomed Optics Express. (2015) 6:3701–13. doi: 10.1364/BOE.6.003701, PMID: 26504622 PMC4605031

[130] Chen S Shu X Nesper PL Liu W Fawzi AA Zhang HF . Retinal oximetry in humans using visible-light optical coherence tomography. Biomed Optics Express. (2017) 8:1415–29. doi: 10.1364/BOE.8.001415, PMID: 28663838 PMC5480553

[131] Song W Shao W Yi W Liu R Desai M Ness S . Visible light optical coherence tomography angiography (vis-OCTA) facilitates local microvascular oximetry in the human retina. Biomed Optics Express. (2020) 11:4037–51. doi: 10.1364/BOE.395843, PMID: 33014584 PMC7510897

[132] Apelian C Harms F Thouvenin O Boccara AC . Dynamic full field optical coherence tomography: subcellular metabolic contrast revealed in tissues by interferometric signals temporal analysis. Biomed Optics Express. (2016) 7:1511–24. doi: 10.1364/BOE.7.001511, PMID: 27446672 PMC4929658

[133] Ren C Hao S Wang F Matt A Amaral MM Yang D . Dynamic contrast optical coherence tomography (DyC-OCT) for label-free live cell imaging. Commun Biol. (2024) 7:278. doi: 10.1038/s42003-024-05973-5, PMID: 38448627 PMC10918170

[134] Thouvenin O Fink M Boccara C . Dynamic multimodal full-field optical coherence tomography and fluorescence structured illumination microscopy. J Biomed Optics. (2017) 22:026004–4. doi: 10.1117/1.JBO.22.2.026004, PMID: 28195601

[135] Park S Veluvolu V Martin WS Nguyen T Park J Sackett DL . Label-free, non-invasive, and repeatable cell viability bioassay using dynamic full-field optical coherence microscopy and supervised machine learning. Biomed Opt Express. (2022) 13:3187–94. doi: 10.1364/BOE.452471, PMID: 35781969 PMC9208588

[136] Babcock HW . The possibility of compensating astronomical seeing. Publ Astronomical Soc Pac. (1953) 65:229–36. doi: 10.1086/126606

[137] Liang J Williams DR Miller DT . Supernormal vision and high-resolution retinal imaging through adaptive optics. JOSA A. (1997) 14:2884–92. doi: 10.1364/JOSAA.14.002884, PMID: 9379246

[138] Williams D Yoon G-Y Porter J Guirao A Hofer H Cox I . Visual benefit of correcting higher order aberrations of the eye. J Refract Surg. (2000) 16:S554–9. doi: 10.3928/1081-597X-20000901-12, PMID: 11019871

[139] Zawadzki RJ Jones SM Olivier SS Zhao M Bower BA Izatt JA . Adaptive-optics optical coherence tomography for high-resolution and high-speed 3D retinal *in vivo* imaging. Optics Express. (2005) 13:8532–46. doi: 10.1364/OPEX.13.008532, PMID: 19096728 PMC2605068

[140] Roorda A Romero-Borja F Donnelly IIIWJ Queener H Hebert TJ Campbell MC . Adaptive optics scanning laser ophthalmoscopy. Optics Express. (2002) 10:405–12. doi: 10.1364/OE.10.000405, PMID: 19436374

[141] Palczewska G Dong Z Golczak M Hunter JJ Williams DR Alexander NS . Noninvasive two-photon microscopy imaging of mouse retina and retinal pigment epithelium through the pupil of the eye. Nat Med. (2014) 20:785–9. doi: 10.1038/nm.3590, PMID: 24952647 PMC4087080

[142] Liang J Grimm B Goelz S Bille JF . Objective measurement of wave aberrations of the human eye with the use of a Hartmann–Shack wave-front sensor. JOSA A. (1994) 11:1949–57. doi: 10.1364/JOSAA.11.001949, PMID: 8071736

[143] Hayashi A Nakamura T Otsuka M Miyakoshi A Oiwake T Ueda T . Observation of microcystic changes in the inner retina with adaptive optics fundus camera. Invest Ophthalmol Visual Sci. (2014) 55:2608–8. doi: 10.1364/JOSAA.11.001949, PMID: 8071736

[144] Popovic Z Knutsson P Thaung J Owner-Petersen M Sjöstrand J . Noninvasive imaging of human foveal capillary network using dual-conjugate adaptive optics. Invest Ophthalmol Visual Sci. (2011) 52:2649–55. doi: 10.1167/iovs.10-6054, PMID: 21228372

[145] Sulai YN Dubra A . Adaptive optics scanning ophthalmoscopy with annular pupils. Biomed Optics Express. (2012) 3:1647–61. doi: 10.1364/BOE.3.001647, PMID: 22808435 PMC3395488

[146] Burns S . Applications of adaptive optics scanning laser ophthalmoscopes. J Vision. (2017) 17:25–5. doi: 10.1167/17.15.25a

[147] Hermann B Fernández E Unterhuber A Sattmann H Fercher A Drexler W . Adaptive-optics ultrahigh-resolution optical coherence tomography. Optics Lett. (2004) 29:2142–4. doi: 10.1364/OL.29.002142, PMID: 15460883

[148] Salas M Augustin M Ginner L Kumar A Baumann B Leitgeb R . Visualization of micro-capillaries using optical coherence tomography angiography with and without adaptive optics. Biomed Optics Express. (2017) 8:207–22. doi: 10.1364/BOE.8.000207, PMID: 28101412 PMC5231293

[149] Zhang P Wahl DJ Mocci J Miller EB Bonora S Sarunic MV . Adaptive optics scanning laser ophthalmoscopy and optical coherence tomography (AO-SLO-OCT) system for *in vivo* mouse retina imaging. BioMed Opt Express. (2023) 14:299–314. doi: 10.1364/BOE.473447, PMID: 36698677 PMC9841993

[150] Zhou M Zhang Y Karimi Monsefi A Choi SS Doble N Parthasarathy S . Reducing manual labeling requirements and improved retinal ganglion cell identification in 3D AO-OCT volumes using semi-supervised learning. Biomed Opt Express. (2024) 15:4540–56. doi: 10.1364/BOE.526053, PMID: 39346977 PMC11427208

[151] Das V Zhang F Bower AJ Li J Liu T Aguilera N . Revealing speckle obscured living human retinal cells with artificial intelligence assisted adaptive optics optical coherence tomography. Commun Med. (2024) 4:68. doi: 10.1038/s43856-024-00483-1, PMID: 38600290 PMC11006674

[152] Duncan JL Zhang Y Gandhi J Nakanishi C Othman M Branham KE . High-resolution imaging with adaptive optics in patients with inherited retinal degeneration. Invest Ophthalmol Visual Sci. (2007) 48:3283–91. doi: 10.1167/iovs.06-1422, PMID: 17591900

[153] Wolfing JI Chung M Carroll J Roorda A Williams DR . High-resolution retinal imaging of cone–rod dystrophy. Ophthalmology. (2006) 113:1014–1019. e1011. doi: 10.1016/j.ophtha.2006.01.056, PMID: 16650474

[154] Syed R Sundquist SM Ratnam K Zayit-Soudry S Zhang Y Crawford JB . High-resolution images of retinal structure in patients with choroideremia. Invest Ophthalmol Visual Sci. (2013) 54:950–61. doi: 10.1167/iovs.12-10707, PMID: 23299470 PMC3564452

[155] Werner JS Keltner JL Zawadzki R Choi S . Outer retinal abnormalities associated with inner retinal pathology in nonglaucomatous and glaucomatous optic neuropathies. Eye. (2011) 25:279–89. doi: 10.1038/eye.2010.218, PMID: 21293495 PMC3071640

[156] Helmchen F Denk W . Deep tissue two-photon microscopy. Nat Methods. (2005) 2:932–40. doi: 10.1038/nmeth818, PMID: 16299478

[157] Göppert-Mayer M . Über Elementarakte mit zwei Quantensprüngen. Annalen Der Physik. (1931) 401:273–94. doi: 10.1002/andp.19314010303

[158] Franken P Hill AE Peters Ce Weinreich G . Generation of optical harmonics. Phys Rev Lett. (1961) 7:118. doi: 10.1103/PhysRevLett.7.118

[159] Kaiser W Garrett C . Two-photon excitation in ca F 2: eu 2 +. Phys Rev Lett. (1961) 7:229. doi: 10.1103/PhysRevLett.7.229

[160] Denk W Strickler JH Webb WW . Two-photon laser scanning fluorescence microscopy. Science. (1990) 248:73–6. doi: 10.1126/science.2321027, PMID: 2321027

[161] Theer P Hasan MT Denk W . Two-photon imaging to a depth of 1000 µm in living brains by use of a Ti: Al 2 O 3 regenerative amplifier. Optics Lett. (2003) 28:1022–4. doi: 10.1364/OL.28.001022, PMID: 12836766

[162] Oheim M Beaurepaire E Chaigneau E Mertz J Charpak S . Two-photon microscopy in brain tissue: parameters influencing the imaging depth. J Neurosci Methods. (2001) 111:29–37. doi: 10.1016/S0165-0270(01)00438-1, PMID: 11574117

[163] Beaurepaire E Mertz J . Epifluorescence collection in two-photon microscopy. Appl Optics. (2002) 41:5376–82. doi: 10.1364/AO.41.005376, PMID: 12211567

[164] Patterson GH Piston DW . Photobleaching in two-photon excitation microscopy. Biophys J. (2000) 78:2159–62. doi: 10.1016/S0006-3495(00)76762-2, PMID: 10733993 PMC1300807

[165] Hopt A Neher E . Highly nonlinear photodamage in two-photon fluorescence microscopy. Biophys J. (2001) 80:2029–36. doi: 10.1016/S0006-3495(01)76173-5, PMID: 11259316 PMC1301392

[166] Helmchen F Svoboda K Denk W Tank DW . *In vivo* dendritic calcium dynamics in deep-layer cortical pyramidal neurons. Nat Neurosci. (1999) 2:989–96. doi: 10.1038/14788, PMID: 10526338

[167] Squirrell JM Wokosin DL White JG Bavister BD . Long-term two-photon fluorescence imaging of mammalian embryos without compromising viability. Nat Biotechnol. (1999) 17:763–7. doi: 10.1038/11698, PMID: 10429240 PMC5087329

[168] Imanishi Y Batten ML Piston DW Baehr W Palczewski K . Noninvasive two-photon imaging reveals retinyl ester storage structures in the eye. J Cell Biol. (2004) 164:373–83. doi: 10.1083/jcb.200311079, PMID: 14745001 PMC1360214

[169] Maeda A Palczewska G Golczak M Kohno H Dong Z Maeda T . Two-photon microscopy reveals early rod photoreceptor cell damage in light-exposed mutant mice. Proc Natl Acad Sci. (2014) 111:E1428–37. doi: 10.1073/pnas.1317986111, PMID: 24706832 PMC3986171

[170] Schwarz C Sharma R Fischer WS Chung M Palczewska G Palczewski K . Safety assessment in macaques of light exposures for functional two-photon ophthalmoscopy in humans. Biomed Optics Express. (2016) 7:5148–69. doi: 10.1364/BOE.7.005148, PMID: 28018732 PMC5175559

[171] Palczewska G Maeda T Imanishi Y Sun W Chen Y Williams DR . Noninvasive multiphoton fluorescence microscopy resolves retinol and retinal condensation products in mouse eyes. Nat Med. (2010) 16:1444–9. doi: 10.1038/nm.2260, PMID: 21076393 PMC3057900

[172] Boettner EA Wolter JR . Transmission of the ocular media. Invest Ophthalmol Vis Sci. (1962) 1(6):776–83.

[173] So PT Dong CY Masters BR Berland KM . Two-photon excitation fluorescence microscopy. Annu Rev Biomed Eng. (2000) 2:399–429. doi: 10.1146/annurev.bioeng.2.1.399, PMID: 11701518

[174] Palczewska G Stremplewski P Suh S Alexander N Salom D Dong Z . Two-photon imaging of the mammalian retina with ultrafast pulsing laser. JCI Insight. (2018) 3:e121555. doi: 10.1172/jci.insight.121555, PMID: 30185665 PMC6171813

[175] Ávila FJ Gambín A Artal P Bueno JM . *In vivo* two-photon microscopy of the human eye. Sci Rep. (2019) 9:10121. doi: 10.1038/s41598-019-46568-z, PMID: 31300680 PMC6626016

[176] Delori FC Webb RH Sliney DH . Maximum permissible exposures for ocular safety (ANSI 2000), with emphasis on ophthalmic devices. JOSA A. (2007) 24:1250–65. doi: 10.1364/JOSAA.24.001250, PMID: 17429471

[177] Wahl DJ Ju MJ Jian Y Sarunic MV . Non-invasive cellular-resolution retinal imaging with two-photon excited fluorescence. Biomed Optics Express. (2019) 10:4859–73. doi: 10.1364/BOE.10.004859, PMID: 31565530 PMC6757458

[178] Alexander NS Palczewska G Stremplewski P Wojtkowski M Kern TS Palczewski K . Image registration and averaging of low laser power two-photon fluorescence images of mouse retina. Biomed Optics Express. (2016) 7:2671–91. doi: 10.1364/BOE.7.002671, PMID: 27446697 PMC4948621

[179] Siedentopf H Zsigmondy R . Uber sichtbarmachung und größenbestimmung ultramikoskopischer teilchen, mit besonderer anwendung auf goldrubingläser. Annalen Der Physik. (1902) 315:1–39. doi: 10.1002/andp.19023150102

[180] Voie AH Burns D Spelman F . Orthogonal-plane fluorescence optical sectioning: Three-dimensional imaging of macroscopic biological specimens. J Microscopy. (1993) 170:229–36. doi: 10.1111/j.1365-2818.1993.tb03346.x, PMID: 8371260

[181] Huisken J Swoger J Del Bene F Wittbrodt J Stelzer EH . Optical sectioning deep inside live embryos by selective plane illumination microscopy. Science. (2004) 305:1007–9. doi: 10.1126/science.1100035, PMID: 15310904

[182] Keller PJ Schmidt AD Wittbrodt J Stelzer EH . Reconstruction of zebrafish early embryonic development by scanned light sheet microscopy. science. (2008) 322:1065–9. doi: 10.1126/science.1162493, PMID: 18845710

[183] Santi PA . Light sheet fluorescence microscopy: a review. J Histochem Cytochem. (2011) 59:129–38. doi: 10.1369/0022155410394857, PMID: 21339178 PMC3201139

[184] Huisken J Swoger J Lindek S Stelzer EH . @ in Handbook of biological confocal microscopy. Princeton, NJ: Springer (2006) p. 672–9.

[185] Keller PJ Schmidt AD Wittbrodt J Stelzer EH . Digital scanned laser light-sheet fluorescence microscopy (DSLM) of zebrafish and Drosophila embryonic development. Cold Spring Harbor Protoc. (2011), pdb. prot065839. doi: 10.1101/pdb.prot065839, PMID: 21969622

[186] Reynaud EG Peychl J Huisken J Tomancak P . Guide to light-sheet microscopy for adventurous biologists. Nat Methods. (2015) 12:30–4. doi: 10.1038/nmeth.3222, PMID: 25549268

[187] Ji N . Adaptive optical fluorescence microscopy. Nat Methods. (2017) 14:374–80. doi: 10.1038/nmeth.4218, PMID: 28362438

[188] Hubert A Harms F Juvénal R Treimany P Levecq X Loriette V . Adaptive optics light-sheet microscopy based on direct wavefront sensing without any guide star. Opt Lett. (2019) 44:2514–7. doi: 10.1364/OL.44.002514, PMID: 31090720

[189] Luo X Salgueiro Y Beckerman SR Lemmon VP Tsoulfas P Park KK . Three-dimensional evaluation of retinal ganglion cell axon regeneration and pathfinding in whole mouse tissue after injury. Exp Neurol. (2013) 247:653–62. doi: 10.1016/j.expneurol.2013.03.001, PMID: 23510761 PMC3726550

[190] Icha J Schmied C Sidhaye J Tomancak P Preibisch S Norden C . Using light sheet fluorescence microscopy to image zebrafish eye development. JoVE (Journal Visualized Experiments). (2016):e53966. doi: 10.3791/53966, PMID: 27167079 PMC4941907

[191] Prahst C Ashrafzadeh P Mead T Figueiredo A Chang K Richardson D . Mouse retinal cell behaviour in space and time using light sheet fluorescence microscopy. Elife. (2020) 9:e49779. doi: 10.7554/eLife.49779.sa2, PMID: 32073398 PMC7162655

[192] Hangai M Yamamoto M Sakamoto A Yoshimura N . Ultrahigh-resolution versus speckle noise-reduction in spectral-domain optical coherence tomography. Opt Express. (2009) 17:4221–35. doi: 10.1364/OE.17.004221, PMID: 19259257

[193] Carroll J Kay DB Scoles D Dubra A Lombardo M . Designing visible-light optical coherence tomography towards clinics. Quant Imaging Med Surg. (2019) 9:769. doi: 10.21037/qims.2019.05.01, PMID: 31281773 PMC6571199

[194] Fu T-M Liu G Milkie DE Ruan X Görlitz F Shi Y . High-resolution imaging of the retinal nerve fiber layer in normal eyes using adaptive optics scanning laser ophthalmoscopy. PloS One. (2012) 7:e33158. doi: 10.1371/journal.pone.0033158, PMID: 22427978 PMC3299751

[195] Miller D Kocaoglu O Wang Q Lee S . Adaptive optics and the eye (super resolution OCT). Eye. (2011) 25:321–30. doi: 10.1038/eye.2011.1, PMID: 21390066 PMC3113555

[196] Han L Tan B Hosseinaee Z Chen LK Hileeto D Bizheva K . Three dimensional two-photon brain imaging in freely moving mice using a miniature fiber coupled microscope with active axial-scanning. Sci Rep. (2018) 8:1–14. doi: 10.1038/s41598-018-26326-3, PMID: 29802371 PMC5970169

[197] Liu A Xiao W Li R Liu L Chen L . Adaptive optics two-photon microscopy enables near-diffraction-limited and functional retinal imaging *in vivo*. Light: Sci Appl. (2020) 9:1–11. doi: 10.1038/s41377-020-0317-9, PMID: 32411364 PMC7203252

[198] Liu A Xiao W Li R Liu L Chen L . Comparison of optical projection tomography and light-sheet fluorescence microscopy. J Microscopy. (2019) 275:3–10. doi: 10.1111/jmi.12796, PMID: 31012490

[199] Schuman JS Fujimoto JG Duker J Ishikawa H . Optical coherence tomography of ocular diseases. Boca Raton, FL: CRC Press (2024).

[200] Carroll J Kay DB Scoles D Dubra A Lombardo M . Adaptive optics retinal imaging–clinical opportunities and challenges. Curr Eye Res. (2013) 38:709–21. doi: 10.3109/02713683.2013.784792, PMID: 23621343 PMC4031042

[201] Takayama K Ooto S Hangai M Arakawa N Oshima S Shibata N . A multimodal adaptive optical microscope for *in vivo* imaging from molecules to organisms. bioRxiv. (2025). doi: 10.1371/journal.pone.0033158, PMID: 40661351

[202] Wieser W Biedermann BR Klein T Eigenwillig CM Huber R . Multi-megahertz OCT: High quality 3D imaging at 20 million A-scans and 4.5 GVoxels per second. Optics Express. (2010) 18:14685–704. doi: 10.1364/OE.18.014685, PMID: 20639955

[203] Hangai M Yamamoto M Sakamoto A Yoshimura N . Line-scanning SD-OCT for *in-vivo*, non-contact, volumetric, cellular resolution imaging of the human cornea and limbus. Biomed Optics Express. (2022) 13:4007–20. doi: 10.1364/BOE.465916, PMID: 35991928 PMC9352278

[204] Morgan JI Vergilio GK Hsu J Dubra A Cooper RF . The reliability of cone density measurements in the presence of rods. Trans Vision Sci Technol. (2018) 7:21–1. doi: 10.1167/tvst.7.3.21, PMID: 29946495 PMC6016505

[205] Geng Y Dubra A Yin L Merigan WH Sharma R Libby RT . Adaptive optics retinal imaging in the living mouse eye. Biomed Optics Express. (2012) 3:715–34. doi: 10.1364/BOE.3.000715, PMID: 22574260 PMC3345801

[206] Jonnal RS Kocaoglu OP Zawadzki RJ Liu Z Miller DT Werner JS . A review of adaptive optics optical coherence tomography: technical advances, scientific applications, and the future. Invest Ophthalmol Visual Sci. (2016) 57:OCT51–68. doi: 10.1167/iovs.16-19103, PMID: 27409507 PMC4968917

[207] Pircher M Zawadzki RJ . Review of adaptive optics OCT (AO-OCT): principles and applications for retinal imaging. Biomed Optics Express. (2017) 8:2536–62. doi: 10.1364/BOE.8.002536, PMID: 28663890 PMC5480497

[208] Zhang F Kurokawa K Lassoued A Crowell JA Miller DT . Cone photoreceptor classification in the living human eye from photostimulation-induced phase dynamics. Proc Natl Acad Sci. (2019) 116:7951–6. doi: 10.1073/pnas.1816360116, PMID: 30944223 PMC6475411

[209] Roorda A Williams DR . The arrangement of the three cone classes in the living human eye. Nature. (1999) 397:520–2. doi: 10.1038/17383, PMID: 10028967

[210] Litts KM Cooper RF Duncan JL Carroll J . Photoreceptor-based biomarkers in AOSLO retinal imaging. Invest Ophthalmol Visual Sci. (2017) 58:BIO255–67. doi: 10.1167/iovs.17-21868, PMID: 28873135 PMC5584616

[211] Roorda A . Applications of adaptive optics scanning laser ophthalmoscopy. Optomet Vision Sci. (2010) 87:260. doi: 10.1097/OPX.0b013e3181d39479, PMID: 20160657 PMC2911957

[212] Zhang B Li N Kang J He Y Chen X-M . Adaptive optics scanning laser ophthalmoscopy in fundus imaging, a review and update. Int J Ophthalmol. (2017) 10:1751. doi: 10.18240/ijo.2017.11.18, PMID: 29181321 PMC5686376

[213] Pircher M Zawadzki RJ . Combining adaptive optics with optical coherence tomography: Unveiling the cellular structure of the human retina *in vivo*. Expert Rev Ophthalmol. (2007) 2:1019–35. doi: 10.1586/17469899.2.6.1019

[214] Scoles D Sulai YN Langlo CS Fishman GA Curcio CA Carroll J . *In vivo* imaging of human cone photoreceptor inner segments. Invest Ophthalmol Visual Sci. (2014) 55:4244–51. doi: 10.1167/iovs.14-14542, PMID: 24906859 PMC4095721

[215] Azimipour M Valente D Vienola KV Werner JS Zawadzki RJ Jonnal RS . Optoretinogram: optical measurement of human cone and rod photoreceptor responses to light. Optics Lett. (2020) 45:4658–61. doi: 10.1364/OL.398868, PMID: 32870829 PMC7891461

[216] Tan B Li H Zhuo Y Han L Mupparapu R Nanni D . Light-evoked deformations in rod photoreceptors, pigment epithelium and subretinal space revealed by prolonged and multilayered optoretinography. Nat Commun. (2024) 15:5156. doi: 10.1038/s41467-024-49014-5, PMID: 38898002 PMC11186825

[217] Lee B Jeong S Lee J Kim TS Braaf B Vakoc BJ . Wide-field three-dimensional depth-invariant cellular-resolution imaging of the human retina. Small. (2023) 19:e2203357. doi: 10.1002/smll.202203357, PMID: 36642824 PMC10023497

[218] Cooper RF Brainard DH Morgan JI . Optoretinography of individual human cone photoreceptors. Opt Express. (2020) 28:39326–39. doi: 10.1364/OE.409193, PMID: 33379485 PMC7771891

[219] Pandiyan VP Jiang X Maloney-Bertelli A Kuchenbecker JA Sharma U Sabesan R . High-speed adaptive optics line-scan OCT for cellular-resolution optoretinography. Biomed Optics Express. (2020) 11:5274–96. doi: 10.1364/BOE.399034, PMID: 33014614 PMC7510866

[220] Son T Kim T-H Ma G Kim H Yao X . Functional intrinsic optical signal imaging for objective optoretinography of human photoreceptors. Exp Biol Med. (2021) 246:639–43. doi: 10.1177/1535370220978898, PMID: 33307802 PMC7988726

[221] Vienola KV Valente D Zawadzki RJ Jonnal RS . Velocity-based optoretinography for clinical applications. Optica. (2022) 9:1100–8. doi: 10.1364/OPTICA.460835, PMID: 40161254 PMC11951274

[222] Lassoued A Zhang F Kurokawa K Liu Y Bernucci MT Crowell JA . Cone photoreceptor dysfunction in retinitis pigmentosa revealed by optoretinography. Proc Natl Acad Sci. (2021) 118:e2107444118. doi: 10.1073/pnas.2107444118, PMID: 34795055 PMC8617487

[223] Gofas-Salas E Rui Y Mecê P Zhang M Snyder VC Vienola KV . Design of a radial multi-offset detection pattern for *in vivo* phase contrast imaging of the inner retina in humans. Biomed Optics Express. (2022) 13:117–32. doi: 10.1364/BOE.441808, PMID: 35154858 PMC8803027

[224] Patterson EJ Kalitzeos A Kane TM Singh N Kreis J Pennesi ME . Foveal cone structure in patients with blue cone monochromacy. Invest Ophthalmol Visual Sci. (2022) 63:23–3. doi: 10.1167/iovs.63.11.23, PMID: 36301530 PMC9624264

[225] Heitkotter H Patterson EJ Woertz EN Cava JA Gaffney M Adhan I . Extracting spacing-derived estimates of rod density in healthy retinae. Biomed Optics Express. (2023) 14:1–17. doi: 10.1364/BOE.473101, PMID: 36698662 PMC9842010

[226] Mulligan JB MacLeod DI Statler IC . @ in Vision Science and Its Application Topical Meeting. Santa Fe, NM: NASA (1994).

[227] Wells-Gray EM Choi SS Zawadzki RJ Finn SC Greiner CA Werner JS . Volumetric imaging of rod and cone photoreceptor structure with a combined adaptive optics-optical coherence tomography-scanning laser ophthalmoscope. J Biomed Optics. (2018) 23:036003. doi: 10.1117/1.JBO.23.3.036003, PMID: 29508564 PMC8357331

[228] Bernucci M Kurokawa K Liu Y Zhang F Crowell J Miller DT . Spectrally resolving S, M, and L cone sensitivities across the visible spectrum using AO-OCT optoretinography with a supercontinuum laser. Invest Ophthalmol Visual Sci. (2022) 63:397–F0435-0397–F0435.

[229] Ma G Son T Kim TH Yao X . *In vivo* optoretinography of phototransduction activation and energy metabolism in retinal photoreceptors. J Biophoton. (2021) 14:e202000462. doi: 10.1002/jbio.202000462, PMID: 33547871 PMC8240094

[230] Kim T-H Wang B Lu Y Son T Yao X . Functional optical coherence tomography enables *in vivo* optoretinography of photoreceptor dysfunction due to retinal degeneration. Biomed Optics Express. (2020) 11:5306–20. doi: 10.1364/BOE.399334, PMID: 33014616 PMC7510876

[231] Roorda A . Optoretinography is coming of age. Proc Natl Acad Sci. (2021) 118:e2119737118. doi: 10.1073/pnas.2119737118, PMID: 34907020 PMC8713980

[232] Jiang C Wang W Ling N Xu G Rao X Li X . High-resolution imaging of living retina through optic adaptive retinal imaging system. Yan Ke Xue Bao (2016). (2002) 18:131–5., PMID: 15510740

[233] Liu Z Kurokawa K Zhang F Lee JJ Miller DT . Imaging and quantifying ganglion cells and other transparent neurons in the living human retina. Proc Natl Acad Sci. (2017) 114:12803–8. doi: 10.1073/pnas.1711734114, PMID: 29138314 PMC5715765

[234] Zhang P Miller EB Manna SK Meleppat RK Pugh EN Zawadzki R . Temporal speckle-averaging of optical coherence tomography volumes for *in-vivo* cellular resolution neuronal and vascular retinal imaging. Neurophotonics. (2019) 6:041105. doi: 10.1117/1.NPh.6.4.041105, PMID: 31528657 PMC6732665

[235] Schroeter EH Wong RO Gregg RG . *In vivo* development of retinal ON-bipolar cell axonal terminals visualized in nyx:: MYFP transgenic zebrafish. Visual Neurosci. (2006) 23:833–43. doi: 10.1017/S0952523806230219, PMID: 17020638

[236] Lu Q Ganjawala T Ivanova E Cheng J Troilo D Pan Z . AAV-mediated transduction and targeting of retinal bipolar cells with improved mGluR6 promoters in rodents and primates. Gene Ther. (2016) 23:680–9. doi: 10.1038/gt.2016.42, PMID: 27115727 PMC4863234

[237] Wang Z McCracken S Williams PR . Transpupillary Two-photon *in vivo* imaging of the mouse retina. JoVE (Journal Visualized Experiments). (2021):e61970. doi: 10.3791/61970, PMID: 33645555 PMC9385267

[238] Kim TH Ma G Son T Yao X . Functional optical coherence tomography for intrinsic signal optoretinography: recent developments and deployment challenges. Front Med (Lausanne). (2022) 9:864824. doi: 10.3389/fmed.2022.864824, PMID: 35445037 PMC9013890

[239] Masri RA Weltzien F Purushothuman S Lee SC Martin PR Grünert U . Composition of the inner nuclear layer in human retina. Invest Ophthalmol Visual Sci. (2021) 62:22–2. doi: 10.1167/iovs.62.9.22, PMID: 34259817 PMC8288061

[240] Meah A Boodram V Lim H . Imaging unlabeled axons in the mouse retina by second harmonic generation. bioRxiv. (2022). 2022.2005. 2016.492107. doi: 10.1101/2022.05.16.492107

[241] Geng Y Greenberg KP Wolfe R Gray DC Hunter JJ Dubra A . *In vivo* imaging of microscopic structures in the rat retina. Invest Ophthalmol Visual Sci. (2009) 50:5872–9. doi: 10.1167/iovs.09-3675, PMID: 19578019 PMC2873188

[242] Sharma R Yin L Geng Y Merigan WH Palczewska G Palczewski K . *In vivo* two-photon imaging of the mouse retina. Biomed Optics Express. (2013) 4:1285–93. doi: 10.1364/BOE.4.001285, PMID: 24009992 PMC3756587

[243] Yin L Masella B Dalkara D Zhang J Flannery JG Schaffer DV . Imaging light responses of foveal ganglion cells in the living macaque eye. J Neurosci. (2014) 34:6596–605. doi: 10.1523/JNEUROSCI.4438-13.2014, PMID: 24806684 PMC4012315

[244] Cheong SK Strazzeri JM Williams DR Merigan WH . All-optical recording and stimulation of retinal neurons *in vivo* in retinal degeneration mice. PloS One. (2018) 13:e0194947. doi: 10.1371/journal.pone.0194947, PMID: 29596518 PMC5875792

[245] Laforest T Carpentras D Künzi M Kowalczuk L Behar-Cohen F Moser C . A new microscopy for imaging retinal cells. arXiv preprint arXiv:1712.08472. (2017).

[246] Pfäffle C Spahr H Kutzner L Burhan S Hilge F Miura Y . Simultaneous functional imaging of neuronal and photoreceptor layers in living human retina. Optics Lett. (2019) 44:5671–4. doi: 10.1364/OL.44.005671, PMID: 31774751

[247] Zhang P Zam A Jian Y Wang X Li Y Lam KS . *In vivo* wide-field multispectral scanning laser ophthalmoscopy–optical coherence tomography mouse retinal imager: longitudinal imaging of ganglion cells, microglia, and Müller glia, and mapping of the mouse retinal and choroidal vasculature. J Biomed Optics. (2015) 20:126005. doi: 10.1117/1.JBO.20.12.126005, PMID: 26677070 PMC4681314

[248] Swanson WH King BJ Burns SA . Interpreting retinal nerve fiber layer reflectance defects based on presence of retinal nerve fiber bundles. Optomet Vision Sci. (2021) 98:531. doi: 10.1097/OPX.0000000000001690, PMID: 33973913 PMC8132612

[249] Chen MF Chui TY Alhadeff P Rosen RB Ritch R Dubra A . Adaptive optics imaging of healthy and abnormal regions of retinal nerve fiber bundles of patients with glaucoma. Invest Ophthalmol Visual Sci. (2015) 56:674–81. doi: 10.1167/iovs.14-15936, PMID: 25574048 PMC4311778

[250] Hood DC Chen MF Lee D Epstein B Alhadeff P Rosen RB . Confocal adaptive optics imaging of peripapillary nerve fiber bundles: implications for glaucomatous damage seen on circumpapillary OCT scans. Trans Vision Sci Technol. (2015) 4:12–2. doi: 10.1167/tvst.4.2.12, PMID: 25909035 PMC4404969

[251] Jayabalan GS Wu Y-K Bille JF Kim S Mao XW Gimbel HV . *In vivo* two-photon imaging of retina in rabbits and rats. Exp Eye Res. (2018) 166:40–8. doi: 10.1016/j.exer.2017.04.009, PMID: 28483661

[252] Jian Y Zawadzki RJ Sarunic MV . Adaptive optics optical coherence tomography for *in vivo* mouse retinal imaging. J Biomed Optics. (2013) 18:056007. doi: 10.1117/1.JBO.18.5.056007, PMID: 23644903 PMC4023643

[253] Miller DA Grannonico M Liu M Kuranov RV Netland PA Liu X . Visible-light optical coherence tomography fibergraphy for quantitative imaging of retinal ganglion cell axon bundles. Trans Vision Sci Technol. (2020) 9:11–1. doi: 10.1167/tvst.9.11.11, PMID: 33110707 PMC7552935

[254] Rubinoff I Beckmann L Wang Y Fawzi AA Liu X Tauber J . Speckle reduction in visible-light optical coherence tomography using scan modulation. Neurophotonics. (2019) 6:041107. doi: 10.1117/1.NPh.6.4.041107, PMID: 31482105 PMC6718816

[255] Roorda A Zhang Y Duncan JL . High-resolution *in vivo* imaging of the RPE mosaic in eyes with retinal disease. Invest Ophthalmol Visual Sci. (2007) 48:2297–303. doi: 10.1167/iovs.06-1450, PMID: 17460294

[256] Morgan JI Dubra A Wolfe R Merigan WH Williams DR . *In vivo* autofluorescence imaging of the human and macaque retinal pigment epithelial cell mosaic. Invest Ophthalmol Visual Sci. (2009) 50:1350–9. doi: 10.1167/iovs.08-2618, PMID: 18952914 PMC2790524

[257] Scoles D Sulai YN Dubra A . *In vivo* dark-field imaging of the retinal pigment epithelium cell mosaic. Biomed Optics Express. (2013) 4:1710–23. doi: 10.1364/BOE.4.001710, PMID: 24049692 PMC3771842

[258] Liu Z Kocaoglu OP Miller DT . 3D imaging of retinal pigment epithelial cells in the living human retina. Invest Ophthalmol Visual Sci. (2016) 57:OCT533–43. doi: 10.1167/iovs.16-19106, PMID: 27472277 PMC4970801

[259] Liu T Jung H Liu J Droettboom M Tam J . Noninvasive near infrared autofluorescence imaging of retinal pigment epithelial cells in the human retina using adaptive optics. Biomed Optics Express. (2017) 8:4348–60. doi: 10.1364/BOE.8.004348, PMID: 29082069 PMC5654784

[260] Tam J Liu J Dubra A Fariss R . *In vivo* imaging of the human retinal pigment epithelial mosaic using adaptive optics enhanced indocyanine green ophthalmoscopy. Invest Ophthalmol Visual Sci. (2016) 57:4376–84. doi: 10.1167/iovs.16-19503, PMID: 27564519 PMC5015921

[261] Schmitz-Valckenberg S Pfau M Fleckenstein M Staurenghi G Sparrow JR Bindewald-Wittich A . Fundus autofluorescence imaging. Prog Retinal Eye Res. (2021) 81:100893. doi: 10.1016/j.preteyeres.2020.100893, PMID: 32758681 PMC12906268

[262] Meleppat RK Ronning KE Karlen SJ Burns ME Pugh EN Zawadzki RJ . *In vivo* multimodal retinal imaging of disease-related pigmentary changes in retinal pigment epithelium. Sci Rep. (2021) 11:1–14. doi: 10.1038/s41598-021-95320-z, PMID: 34376700 PMC8355111

[263] Gao W Cense B Zhang Y Jonnal RS Miller DT . Measuring retinal contributions to the optical Stiles-Crawford effect with optical coherence tomography. Optics Express. (2008) 16:6486–501. doi: 10.1364/OE.16.006486, PMID: 18516251 PMC2405946

[264] Sharma R Williams DR Palczewska G Palczewski K Hunter JJ . Two-photon autofluorescence imaging reveals cellular structures throughout the retina of the living primate eye. Invest Ophthalmol Visual Sci. (2016) 57:632–46. doi: 10.1167/iovs.15-17961, PMID: 26903224 PMC4771181

[265] Liu Z Kurokawa K Hammer DX Miller DT . *In vivo* measurement of organelle motility in human retinal pigment epithelial cells. Biomed Optics Express. (2019) 10:4142–58. doi: 10.1364/BOE.10.004142, PMID: 31453000 PMC6701538

[266] Wahl DJ Jian Y Bonora S Zawadzki RJ Sarunic MV . Wavefront sensorless adaptive optics fluorescence biomicroscope for *in vivo* retinal imaging in mice. Biomed Optics Express. (2016) 7:1–12. doi: 10.1364/BOE.7.000001, PMID: 26819812 PMC4722895

[267] Zawadzki RJ Zhang P Zam A Miller EB Goswami M Wang X . Adaptive-optics SLO imaging combined with widefield OCT and SLO enables precise 3D localization of fluorescent cells in the mouse retina. Biomed Optics Express. (2015) 6:2191–210. doi: 10.1364/BOE.6.002191, PMID: 26114038 PMC4473753

[268] Castanos MV Zhou DB Linderman RE Allison R Milman T Carroll J . Imaging of macrophage-like cells in living human retina using clinical OCT. Invest Ophthalmol Visual Sci. (2020) 61:48–8. doi: 10.1167/iovs.61.6.48, PMID: 32574351 PMC7416910

[269] Alt C Lin CP . @ in ophthalmic technologies XXII Vol. 8209. Bellingham, WA: SPIE (2012) p. 11–9.

[270] Alt C Runnels JM Mortensen LJ Zaher W Lin CP . *In vivo* imaging of microglia turnover in the mouse retina after ionizing radiation and dexamethasone treatment. Invest Ophthalmol Visual Sci. (2014) 55:5314–9. doi: 10.1167/iovs.14-14254, PMID: 25082884

[271] Eter N Engel DR Meyer L Helb H-M Roth F Maurer J . *In vivo* visualization of dendritic cells, macrophages, and microglial cells responding to laser-induced damage in the fundus of the eye. Invest Ophthalmol Visual Sci. (2008) 49:3649–58. doi: 10.1167/iovs.07-1322, PMID: 18316698

[272] Paques M Simonutti M El Mathari B Sahel J-A . *In vivo* observation of the locomotion of microglial cells in the retina of wild-type rodents. Invest Ophthalmol Visual Sci. (2010) 51:5347–7., PMID: 20578032 10.1002/glia.21037

[273] Mezu-Ndubuisi OJ Macke EL Kalavacherla R Nwaba AA Suscha A Zaitoun IS . Long-term evaluation of retinal morphology and function in a mouse model of oxygen-induced retinopathy. Mol Vision. (2020) 26:257., PMID: 32256029 PMC7127927

[274] Kurokawa K Crowell JA Zhang F Miller DT . Suite of methods for assessing inner retinal temporal dynamics across spatial and temporal scales in the living human eye. Neurophotonics. (2020) 7:015013. doi: 10.1117/1.NPh.7.1.015013, PMID: 32206680 PMC7070771

[275] Bosco A Romero CO Breen KT Chagovetz AA Steele MR Ambati BK . Neurodegeneration severity can be predicted from early microglia alterations monitored *in vivo* in a mouse model of chronic glaucoma. Dis Models Mech. (2015) 8:443–55. doi: 10.1242/dmm.018788, PMID: 25755083 PMC4415894

[276] Kumar S Zhuo L . Longitudinal *in vivo* imaging of retinal gliosis in a diabetic mouse model. Exp Eye Res. (2010) 91:530–6. doi: 10.1016/j.exer.2010.07.010, PMID: 20655908

[277] Dulull N Kwa F Osman N Rai U Shaikh B Thrimawithana TR . Recent advances in the management of diabetic retinopathy. Drug Discov Today. (2019) 24:1499–509. doi: 10.1016/j.drudis.2019.03.028, PMID: 30954684

[278] Prasse M Rauscher FG Wiedemann P Reichenbach A Francke M . Optical properties of retinal tissue and the potential of adaptive optics to visualize retinal ganglion cells *in vivo*. Cell Tissue Res. (2013) 353:269–78. doi: 10.1007/s00441-013-1602-1, PMID: 23529360

[279] Franze K Grosche J Skatchkov SN Schinkinger S Foja C Schild D . Müller cells are living optical fibers in the vertebrate retina. Proc Natl Acad Sci. (2007) 104:8287–92. doi: 10.1073/pnas.0611180104, PMID: 17485670 PMC1895942

[280] Kadomoto S Muraoka Y Uji A Ooto S Kawai K Ishikura M . Human foveal cone and müller cells examined by adaptive optics optical coherence tomography. Trans Vision Sci Technol. (2021) 10:17–7. doi: 10.1167/tvst.10.11.17, PMID: 34559184 PMC8475288

[281] Arrigo A Perra C Aragona E Giusto D Doglioni C Pierro L . Extrafoveal Müller cells detection *in vivo* in the human retina: A pilot study based on optical coherence tomography. Exp Eye Res. (2020) 199:108183. doi: 10.1016/j.exer.2020.108183, PMID: 32777210

[282] Szewczuk A Zaleska-Żmijewska A Dziedziak J Szaflik JP . Clinical application of adaptive optics imaging in diagnosis, management, and monitoring of ophthalmological diseases: A narrative review. Med Sci Monit. (2023) 29:e941926. doi: 10.12659/MSM.941926, PMID: 38044597 PMC10704843

[283] Duncan JL Carroll J . Adaptive optics imaging of inherited retinal disease. Cold Spring Harb Perspect Med. (2023) 13:a041285. doi: 10.1101/cshperspect.a041285, PMID: 36220331 PMC10317068

[284] Alexopoulos P Madu C Wollstein G Schuman JS . The development and clinical application of innovative optical ophthalmic imaging techniques. Front Med (Lausanne). (2022) 9:891369. doi: 10.3389/fmed.2022.891369, PMID: 35847772 PMC9279625

[285] Sidiqi A Wahl D Lee S Ma D To E Cui J . *In vivo* retinal fluorescence imaging with curcumin in an Alzheimer mouse model. Front Neurosci. (2020) 14:713. doi: 10.3389/fnins.2020.00713, PMID: 32719582 PMC7350785

[286] Wang J Wang YX Zeng D Zhu Z Li D Liu Y . Artificial intelligence-enhanced retinal imaging as a biomarker for systemic diseases. Theranostics. (2025) 15:3223–33. doi: 10.7150/thno.100786, PMID: 40093903 PMC11905132

[287] Daich Varela M Sen S De Guimaraes TAC Kabiri N Pontikos N Balaskas K . Artificial intelligence in retinal disease: clinical application, challenges, and future directions. Graefes Arch Clin Exp Ophthalmol. (2023) 261:3283–97. doi: 10.1007/s00417-023-06052-x, PMID: 37160501 PMC10169139

[288] Heger KA Waldstein SM . Artificial intelligence in retinal imaging: current status and future prospects. Expert Rev Med Devices. (2024) 21:73–89. doi: 10.1080/17434440.2023.2294364, PMID: 38088362

